# Managing the emergence of pathogen resistance via spatially targeted antimicrobial use

**DOI:** 10.1111/eva.12683

**Published:** 2018-09-26

**Authors:** Kenichi W. Okamoto, David M. Post, David A. Vasseur, Paul E. Turner

**Affiliations:** ^1^ Department of Ecology and Evolutionary Biology Yale University New Haven Connecticut; ^2^ Department of Biology University of St. Thomas Saint Paul Minnesota

**Keywords:** antimicrobial resistance, metapopulation dynamics, spatial heterogeneity, spatial targeting

## Abstract

From agriculture to public health to civil engineering, managing antimicrobial resistance presents a considerable challenge. The dynamics underlying resistance evolution reflect inherently spatial processes. Resistant pathogen strains increase in frequency when a strain that emerges in one locale can spread and replace pathogen subpopulations formerly sensitive to the antimicrobial agent. Moreover, the strength of selection for antimicrobial resistance is in part governed by the extent of antimicrobial use. Thus, altering how antimicrobials are used across a landscape can potentially shift the spatial context governing the dynamics of antimicrobial resistance and provide a potent management tool. Here, we model how the efficacy of adjusting antimicrobial use over space to manage antimicrobial resistance is mediated by competition among pathogen strains and the topology of pathogen metapopulations. For several pathogen migration scenarios, we derive critical thresholds for the spatial extent of antimicrobial use below which resistance cannot emerge, and relate these thresholds to (a) the ability to eradicate antimicrobial‐sensitive pathogens locally and (b) the strength of the trade‐off between resistance ability and competitive performance where antimicrobial use is absent. We find that in metapopulations where patches differ in connectedness, constraining antimicrobial use across space to mitigate resistance evolution only works if the migration of the resistant pathogen is modest; yet, this situation is reversed if the resistant strain has a high colonization rate, with variably connected metapopulations exhibiting less sensitivity to reducing antimicrobial use across space. Furthermore, when pathogens are alternately exposed to sites with and without the antimicrobial, bottlenecking resistant strains through sites without an antimicrobial is only likely to be effective under a strong competition–resistance trade‐off. We therefore identify life‐history constraints that are likely to suggest which pathogens can most effectively be controlled by a spatially targeted antimicrobial regime. We discuss implications of our results for managing and thinking about antimicrobial resistance evolution in spatially heterogeneous contexts.

## 1 INTRODUCTION

Few forms of chemotherapy have had as much impact on human health and welfare as antimicrobial drugs (e.g., Aminov, 2010). Mass antimicrobial administrations either alone or in combination with other intervention strategies play a key role in controlling infectious diseases as diverse as malaria (e.g., Ridley, 2002), HIV (e.g., Perelson, Neumann, Markowitz, Leonard, & Ho, 1996), helminth infections (e.g., Hotez et al., 2008), and influenza (e.g., Burch et al., 2009); antibiotics alone have substantially reduced the global prevalence of previously widely endemic bacterial infections such as tuberculosis (e.g., Castillo‐Chavez & Song, 2004), cholera (e.g., Miller Neilan, Schaefer, Gaff, Fister, & Lenhart, 2010), and trachoma (e.g., Melese et al., 2004). However, antimicrobial resistance is ubiquitous in human‐associated pathogens (Melnyk, Wong, & Kassen, 2015), placing considerable strain on both the global disease burden (WHO, 2014) and key human activities such as agriculture (Silbergeld, Graham, & Price, 2008; Thanner, Drissner, & Walsh, 2016) and sanitation (Bouki, Venieri, & Diamadopoulos, 2013; Lapara et al., 2011; Pruden, Pei, Storteboom, & Carlson, 2006). Managing the emergence and spread of antimicrobial‐resistant pathogens has thus presented a major challenge for several decades (Cohen & Tartasky, 1997; Palumbi, 2001; Spellberg et al., 2008; Walsh, 2003), and there is considerable research on how the prevalence, duration, and extent of local antimicrobial use (e.g., a specific patient or a community of patients) affect the dynamics of antimicrobial resistance in well‐mixed settings (e.g., Baym, Stone, & Kishony, 2016; Bonhoeffer, Lipsitch, & Levin, 1997).

However, the spread of antimicrobial resistance is an inherently spatial process. Resistant pathogen strains rarely, if ever, remain localized for extended periods of time (Laxminarayan et al., 2013), with the dispersal of pathogens being particularly accelerated in increasingly dense transportation networks (Nicolaides, Cueto‐Felgueroso, Gonzalez, & Juanes, 2012; Tatem, Rogers, & Hay, 2006). Moreover, epidemics involving antimicrobial‐resistant strains often involve specific geographic foci (e.g., hospitals and nursing care facilities; CDC, 2013) and the spread of resistant pathogens can track existing infrastructure (e.g., through food processing and distribution channels; Behravesh, Williams, & Tauxe, 2012). These observations suggest that spatially heterogenous antimicrobial applications may play a role in addressing antimicrobial resistance. For instance, if specific, highly trafficked areas are known to be critical to pathogen migration, then targeting antimicrobial use to such sites may present an attractive alternative to widespread application. Despite this potential, we know comparatively little about the prospects of such spatially targeted responses.

The use of spatially targeted strategies to manage resistance evolution has precedent in agriculture. We highlight two examples. Refuge planting, whereby crops not toxic to herbivorous pests are planted at a subset of sites whereas toxic varieties are planted at the remainder of sites, can slow the spread of resistance alleles among crop pest populations (e.g., Gould, 1994). A second spatially oriented strategy aims to construct a heterogeneous landscape of insecticides to constrain the spatial spread of any single pesticide‐resistant allele (Tabashnik, 1994). Although the population genetics of pathogens often differ considerably from those of herbivorous pests, the underlying question in both domains concerns a balancing act: How can the migratory and competitive potential of antimicrobial‐sensitive strains be leveraged to mitigate the strong selective effects of an anthropogenic perturbation (antimicrobial use or agricultural toxins) (e.g., Peck, 2001)?

Despite these parallels, compared to research on antimicrobial resistance in homogenous settings, we are only beginning to understand how altering the spatial structure of a landscape as experienced by a pathogen can drive the emergence and spread of antimicrobial resistance. Smith, Dushoff, Perencevich, Harris, and Levin (2004) describe a multipatch model where hosts can move among sites (e.g., hospitals), and local epidemiological dynamics are described by a susceptible–infectious (SI) model. They calculated how patterns of host movement among discrete sites affect equilibrium antimicrobial resistance frequencies and disease prevalence in the absence of a fitness cost associated with resistance. Smith, Boni, and Laxminarayan (2006) subsequently relaxed the assumption of no fitness costs, numerically evaluating the long‐term prevalence and frequency of antimicrobial resistance across patches. They found the prevalence of antimicrobial resistance in a given patch to depend on how many neighboring patches used antimicrobials. An alternative approach was taken by Débarre, Lenormand, and Gandon (2009), analyzing models in which pathogens disperse along a continuous cline in a host–pathogen system characterized by susceptible–infected–susceptible (SIS) epidemiological dynamics. Débarre et al. (2009) identified how the critical size of the antimicrobially treated region (below which antimicrobial resistance is not viable) shifts in response to dispersal and local epidemiological dynamics.

Because these studies couple local epidemiological dynamics and movement, they require specifying the local compartmental models for analysis (e.g., an SI model in Smith et al., 2004 and an SIS model in Débarre et al., 2009). Thus, generalizing the results concerning spatial antimicrobial resistance management strategies to pathogens with differing natural histories and epidemic compartments can be challenging. Here, we seek to distill the essential dynamics governing the spatial emergence and spread of antimicrobial resistance across diverse local epidemiological contexts. Our aim is twofold. First, we assess spatially based antimicrobial resistance mitigation strategies that restrict antimicrobial use in a generalizable manner to highlight strategies that could be effective across diverse disease systems with distinct local transmission cycles and pathogen life histories. Second, we evaluate how varying the rules that govern local competitive dynamics of pathogens carrying resistant or antimicrobial‐sensitive mutants affects our conclusions. Although the local competitive dynamics among pathogen strains is intricately linked to their interactions with host populations, by decoupling local epidemiological dynamics from spatial selection regimes, we hope to identify approaches to managing antimicrobial resistance over space that apply across systems when only a few basic rules governing local competitive dynamics between antimicrobial‐resistant and antimicrobial‐susceptible strains of a pathogen are known.

## 2 METHODS

### 2.1 Model descriptions

We use a patch‐occupancy model to characterize the pathogen's metapopulation dynamics (Hanski, 1998, 1999; Holyoak & Ray, 1999; Levins, 1969). In the interest of keeping our analyses tractable, we do not explicitly model within‐patch epidemiological dynamics. Rather, these intrapatch processes are modeled to occur on a much faster timescale than the metapopulation's dynamics. Similarly, a sufficient number of suitable hosts are assumed to be available across the metapopulation throughout the duration of analyses.

We model a landscape consisting of discrete sites representing distinct geographic entities (e.g., municipalities, hospitals, farms, or even individual hosts) in which the pathogen can potentially become endemic. As we seek to assess how the use of an antimicrobial across space affects the emergence of antimicrobial resistance, we consider two patch types: patches in which the antimicrobial is routinely used (*a*) and patches in which the antimicrobial is nonexistent (*n*). This distinction enables us to describe situations where certain types of sites may be targeted for antimicrobial use. For instance, if the metapopulation describes an urban community, antimicrobials may only be administered at hospitals or clinics rather than at residences or other workplaces. In this scenario, the frequency of sites *a* that are subject to antimicrobial use could therefore correspond to the frequency of clinics or hospitals among all sites in the community. Whereas all sites either do or do not use an antimicrobial, the pathogen will not necessarily be endemic at all sites. In particular, a pathogen population of strain *i* can go extinct in a given site of type *h *= *n*,* a* at a rate *e*_*h*,*i*_. Such local extinctions can result, for instance, from the pathogen having driven the local host population extinct, the host population temporarily clearing a herd‐immunity threshold (e.g., Fine, 1993), or, when patches describe individual hosts, immunity‐mediated clearing of a pathogen population. We assume that conditions in a site recover sufficiently quickly (e.g., through host demographic turnover or waning immunity) to permit eventual pathogen reestablishment. As is commonly observed (e.g., McDonald & Linde, 2002; but see Awadalla, 2003), we do not consider back‐mutations from antimicrobial‐resistant to antimicrobial‐sensitive strains. Each pathogen strain *i* migrates from endemic to uninfested sites at a rate *c*_*i*_.

We begin by considering a pathogen metapopulation in which pathogens are able to migrate to any unoccupied site from any site in which they are endemic. As a first step, we assume that the antimicrobial‐sensitive strain cannot occupy sites where the antimicrobial is present. In effect, those sites act as refuges for the resistant strain. We denote by *a*_*r*_ the fraction of sites in which an antimicrobial is used and which are occupied by resistant pathogen populations, and define *n*_*i*_ as the fraction of sites where the antimicrobial is not used and a pathogen of strain *i* is endemic. We assume that in sites without the antimicrobial, an invading strain of type *j* replaces a resident of type *i* at a rate *g*_*i*,*j*_. *g*_*i*,*j*_ is a composite parameter representing the integrated effects of migration, establishment, and local competitive replacement. Thus, although the ability of a strain *i* to replace another strain may be related to its ability *c*_*i*_ to occupy empty patches, we assume that the two modes of colonization (the colonization of empty sites and competitive displacement) can be described with two separate parameters. An important implication of this is that the rank order of abilities *c*_*i*_ of strain *i* to occupy empty patches may not necessarily be positively correlated with the rank order of the strain's abilities *g*_*i*,*j*_ to displace other strains. We further characterize the fraction of sites not using the antimicrobial as *f*_*n*_. Table 1 summarizes the state variables and parameters.

**Table 1:** State variables, parameters, and their definitions

Parameter	Interpretation
*n*_*r*_	The fraction of sites without the antimicrobial occupied by the resistant strain
*a*_*r*_	The fraction of sites with the antimicrobial occupied by the resistant strain
*n*_*s*_	The fraction of sites without the antimicrobial occupied by the antimicrobial‐sensitive strain
*a*_*s*_	The fraction of sites with the antimicrobial occupied by the antimicrobial‐sensitive strain
*c*_*i*_	The rate at which pathogen of strain *i* migrates from endemic to uninfested sites
*e*_*i*,*j*_	The rate at which pathogen of strain *j* goes extinct in a site of type *i*
*g*_*i*,*j*_	The rate at which pathogen of strain *j* replaces a pathogen of strain *i* in sites without the antimicrobial
*f*_*n*_	The fraction of sites not subject to antimicrobial application
*k*	A site's degree
$D_{j,i,k,k^{\prime }}$	The propensity of pathogens of strain *i* to successfully migrate from a patch of degree *k*′ to a patch of type *j* with degree *k*
*ϵ*_*j*,*k*_	The fraction of empty sites of degree *k* with (*j* = *a*) or without (*j* = *n*) the antimicrobial
*n*_*i*,*k*_	The fraction of sites with degree *k* without the antimicrobial occupied by strain *i*
*a*_*i*,*k*_	The fraction of sites with degree *k* with the antimicrobial occupied by strain *i*

We model the dynamics of sites occupied by antimicrobial‐sensitive and antimicrobial‐resistant pathogens across the metapopulation as:

(1) $$\begin{array}{cc} \frac{dn_{r}}{dt}= & c_{r}\left(a_{r}+n_{r}\right)\left(f_{n}-n_{s}-n_{r}\right)-e_{n,r}n_{r}-g_{r,s}n_{s}n_{r}+g_{s,r}\left(a_{r}+n_{r}\right)n_{s}\\ \frac{da_{r}}{dt}= & c_{r}\left(a_{r}+n_{r}\right)\left(1-f_{n}-a_{r}\right)-e_{a,r}a_{r}\\ \frac{dn_{s}}{dt}= & c_{s}n_{s}\left(f_{n}-n_{s}-n_{r}\right)-e_{n,s}n_{s}-g_{s,r}\left(a_{r}+n_{r}\right)n_{s}+g_{r,s}n_{s}n_{r}. \end{array}$$

At least on the timescale of landscape‐wide dynamics, Model (1) precludes within‐patch strain coexistence. In principle, our model can also apply to cases where local epidemiological dynamics do not result in all hosts becoming infected in a single patch. Rather, what is important for our model is that within a given patch, hosts that are infected are all infected by a single pathogen strain. We note that sites with antimicrobial use effectively become sinks for the antimicrobial‐sensitive strain. The assumption that within‐patch coexistence among strains cannot occur, at least on the timescale of metapopulation dynamics, may seem like a strong one. Nevertheless, there are several reasons why focusing on situations where only a single strain can occupy a patch may be warranted, at least as a first step. First, simulations suggest that the region of parameter space permitting local coexistence between antimicrobial‐resistant and antimicrobial‐sensitive strains to be smaller than the region permitting competitive displacement of one strain by another (Colijn et al., 2010). Furthermore, although few studies have explored the spread of antimicrobial resistance using a metapopulation framework, the speed at which previous‐generation antimicrobials become clinically ineffective in certain regions or habitats (e.g., Cohen, 1992; Hawkey & Jones, 2009; Okeke & Edelman, 2001; Panlilio et al., 1992; Pearce et al., 2009; Willems et al., 2005) suggests that local competitive displacement of antimicrobial‐sensitive strains by antimicrobial‐resistant strains may be common.

We next extend Model (1) to consider the case where the antimicrobial‐sensitive strain is able to persist in sites where the antimicrobial is used, albeit at relatively lower densities than in sites without the antimicrobial. The persistence of antimicrobial‐sensitive strains in sites where the antimicrobial is used might be reasonable if, for instance, within‐patch spatial heterogeneity provides a handful of refuges to the antimicrobial‐sensitive pathogen, such as when there are reservoir or secondary hosts that do not receive the antimicrobial in such sites. For example, antimicrobials may be used only on specific animals in farms, permitting an antimicrobial‐sensitive strain to persist among alternative hosts in mixed livestock farms (Fessler et al., 2012; Spohr et al., 2011). We assume, however, that antimicrobial‐sensitive strains occupying sites with the antimicrobial are displaced by the antimicrobial‐resistant strain at the resistant strain's colonization rate *c*_*r*_ of empty patches. When antimicrobial‐sensitive strains can persist in sites where the antimicrobial is used, Equations (1) are modified as:

(2) $$\begin{array}{cc} \frac{dn_{r}}{dt}= & c_{r}\left(a_{r}+n_{r}\right)\left(f_{n}-n_{r}-n_{s}\right)-e_{n,r}n_{r}-g_{r,s}\left(a_{s}+n_{s}\right)n_{r}+g_{s,r}\left(a_{r}+n_{r}\right)n_{s}\\ \frac{da_{r}}{dt}= & c_{r}\left(a_{r}+n_{r}\right)\left(\left(1-f_{n}\right)-a_{r}-a_{s}\right)-e_{a,r}a_{r}+c_{r}a_{s}\left(a_{r}+n_{r}\right)\\ \frac{dn_{s}}{dt}= & c_{s}\left(a_{s}+n_{s}\right)\left(f_{n}-n_{r}-n_{s}\right)-e_{n,s}n_{s}-g_{s,r}\left(a_{r}+n_{r}\right)n_{s}+g_{r,s}\left(a_{s}+n_{s}\right)n_{r}\\ \frac{da_{s}}{dt}= & c_{r}\left(a_{s}+n_{s}\right)\left(\left(1-f_{n}\right)-a_{r}-a_{s}\right)-e_{a,s}a_{s}-c_{r}a_{s}\left(a_{r}+n_{r}\right). \end{array}$$

Model (2) characterizes an important asymmetry that arises between the strains in sites where the antimicrobial is available. Namely, a resistant pathogen strain will always displace a resident antimicrobial‐sensitive pathogen strain at such sites, but an invading antimicrobial‐sensitive strain is unable to displace a resident resistant strain where the antimicrobial is used.

We note two distinctions between our approach and some earlier work (e.g., Smith et al. (2006) and Débarre et al. (2009)). First, we focus on the spatial selection pressures in response to antimicrobial use, rather than the coupling of local epidemiological dynamics across space. This allows us to potentially identify strategies that could be expected to apply across diverse disease systems with distinct local transmission cycles and epidemiological dynamics. For example, the local epidemiological dynamics in a hospital resulting from antimicrobial use may differ from the local epidemiological dynamics in an agricultural setting. Our approach therefore enables us to distill the essential dynamics governing the spatial emergence and spread of antimicrobial resistance across different local epidemiological contexts. Second, we explicitly consider pathogen strains that occupy discrete spatial units, rather than modeling a continuous diffusion of the pathogen across the landscape. One advantage of this approach is that it may provide some heuristic correspondence between our model and the distinct geographic entities (cities, farms, clinics) in which pathogen populations are often clustered.

Models (2) and (1) follow a common assumption of discrete‐patch metapopulation models in allowing pathogens to migrate from a patch in which they are endemic to any other patch (e.g., Hanski, 1999; Levins, 1969). When the distinct patches are reinterpreted as individual host organisms, Models (2), (3), (4), (5), (6), (7), (8), (9) can recover a susceptible‐infectious epidemiological model with prophylactic use, cocirculation of prophylactic‐resistant and sensitive strains, demographic turnover, and superinfection (i.e., replacement of one pathogen by another in a single host—e.g., Tanaka & Feldman, 1999; Lipsitch, 1997; Park, Haven, Kaplan, & Gandon, 2015). Nevertheless, because Models (2), (3), (4), (5), (6), (7), (8), (9) can recover well‐mixed models with prophylactic‐resistant pathogens, the resultant dynamics may not be identical to previous models exploring antimicrobial resistance in well‐mixed populations (e.g., Alexander et al., 2007; Austin, Kakehashi, & Anderson, 1997; Bergstrom, Lo, & Lipsitch, 2004; Bonten, Austin, & Lipsitch, 2001; Handel, Longini, & Antia, 2009; Levin et al., 1997; Lipsitch, Cohen, Murray, & Levin, 2007; Lipsitch & Samore, 2002; Moghadas, Bowman, Röst, & Wu, 2008). An important consequence of this is that the dynamics of the spread of antimicrobial resistance in a metapopulation may be more akin to the spread of prophylactic resistance in a well‐mixed population, rather than the spread of antimicrobial resistance per se within a well‐mixed population.

### 2.2 Heterogenous connectivities among patches

The models described above assume that every patch is accessible from every other patch (i.e., they presume an island model of pathogen dispersal; Wright, 1931). These models thus provide a baseline set of predictions against which the spread of antimicrobial resistance in metapopulations with more complex dispersal patterns can be assessed. In the next set of analyses, we ask how the topology of migration between sites affects the relationship between spatially heterogeneous antimicrobial use and the emergence and spread of antimicrobial resistance. We consider two strategies to modeling alternative patch topologies within the metapopulation. First, we model the distribution of the number of migration pathways emanating from patches to be heterogeneous. Second, at a further extreme, we explore what happens when pathogens must migrate through highly connected, centralized hubs to reach other patches. Below, we describe each approach in turn.

In practice, patches within a metapopulation exhibit varying degrees of connectivities to each other (e.g., Xia, Bjørnstad, & Grenfell, 2004). This variability can drive epidemiological dynamics on a metapopulation level. For example, intercountry differences in direct and indirect flights to an original outbreak has been suggested to affect the spatial spread of novel influenza strains (e.g., Hosseini, Sokolow, Vandegrift, Kilpatrick, & Daszak, 2010). To account for distinct connectivities between patches, we consider a metapopulation where each patch is characterized not only by the presence or absence of antimicrobials and the identity of the constituent pathogen strain, but also by the number *k* of other patches linked to it by migration (i.e., in a metapopulation where each patch constitutes a node, the number of edges, or degree associated with each node). The degree *k* of a patch thereby represents the extent of connectivity for that patch. Variable connectivities among patches can then be described through degree heterogeneity.

Following Newman, Strogatz, and Watts (2001) and Colizza and Vespignani (2008), the number of immigrants into a randomly selected patch of degree *k* depends on the patch's degree, the proportion *p*(*k*′ | *k*) of patches of degree *k*′ linked to a patch of degree *k* (with ∑ _*k*_ *p*(*k*′ | *k*) = 1), the fraction of patches of degree *k*′ among all patches, and the propensity $D_{x,i,k,k^{\prime }}$ of pathogens of strain *i* to successfully migrate from a patch of degree *k*′ to a patch of type *x* with degree *k*. The total migration of a pathogen of strain *i* into a patch of degree *k* is therefore $k\sum_{k^{\prime }}p\left(k^{\prime }|k\right)D_{i,k,k^{\prime }}$, where each of the *k* edges will contribute immigrants.

Following Newman, Strogatz, and Watts (2001) and Colizza and Vespignani (2008), the number of immigrants into a randomly selected patch of degree *k* depends on the patch's degree, the proportion *p*(*k*′ | *k*) of patches of degree *k*′ linked to a patch of degree *k* (with ∑ _*k*_ *p*(*k*′ | *k*) = 1), the fraction of patches of degree *k*′ among all patches, and the propensity $D_{x,i,k,k^{\prime }}$ of pathogens of strain *i* to successfully migrate from a patch of degree *k*′ to a patch of type *x* with degree *k*. The total migration of a pathogen of strain *i* into a patch of degree *k* is therefore $k\sum_{k^{\prime }}p\left(k^{\prime }|k\right)D_{i,k,k^{\prime }}$, where each of the *k* edges will contribute immigrants.

We denote by *a*_*i*,*k*_ and *n*_*i*,*k*_ the fraction of sites with and without antibiotic use, respectively, of degree *k* and occupied by strain *i*. We further define the time varying quantity *ϵ*_*m*,*k*_ to describe the fraction of empty sites of degree *k* with (*m* = *a*) or without (*m* = *n*) antimicrobial use. We can then account for heterogeneous connectivity among patches in our metapopulation by modifying Model (2) to track the proportion of patches of degree *k* with and without the antibiotic, and occupied by an antimicrobial‐sensitive or antimicrobial‐resistant strain, as:

(3) $$\begin{array}{cc} \frac{dn_{r,k}}{dt}= & \epsilon_{n,k}k\sum_{k^{\prime }}p\left(k^{\prime }|k\right)D_{\epsilon_{n,k},r,k,k^{\prime }}-e_{n,r}n_{r,k}-n_{r,k}k\sum_{k^{\prime }}p\left(k^{\prime }|k\right)D_{n_{r,k},s,k,k^{\prime }}+n_{s,k}k\sum_{k^{\prime }}p\left(k^{\prime }|k\right)D_{n_{s,k},r,k,k^{\prime }}\\ \frac{da_{r,k}}{dt}= & \epsilon_{a,k}k\sum_{k^{\prime }}p\left(k^{\prime }|k\right)D_{\epsilon_{a,k},r,k,k^{\prime }}-e_{a,r}a_{r,k}+a_{s,k}k\sum_{k^{\prime }}p\left(k^{\prime }|k\right)D_{a_{s,k},r,k,k^{\prime }}\\ \frac{dn_{s,k}}{dt}= & \epsilon_{n,k}k\sum_{k^{\prime }}p\left(k^{\prime }|k\right)D_{\epsilon_{n,k},s,k,k^{\prime }}-e_{n,s}n_{s,k}-n_{s,k}k\sum_{k^{\prime }}p\left(k^{\prime }|k\right)D_{n_{s,k},r,k,k^{\prime }}+n_{r,k}k\sum_{k^{\prime }}p\left(k^{\prime }|k\right)D_{n_{r,k},s,k,k^{\prime }}\\ \frac{da_{s,k}}{dt}= & \epsilon_{a,k}k\sum_{k^{\prime }}p\left(k^{\prime }|k\right)D_{\epsilon_{a,k},s,k,k^{\prime }}-e_{a,s}a_{s,k}-a_{s,k}k\sum_{k^{\prime }}p\left(k^{\prime }|k\right)D_{a_{s,k},r,k,k^{\prime }}. \end{array}$$

We note that Model (3) assumes the local extinction rates of patches is independent of their connectivity. Nevertheless, highly connected patches can have a higher inflow of migrants, resulting in fewer net extinctions than in sparsely connected patches. To maintain continuity with our earlier Models (1) and (2), and to keep our analyses tractable, a consistent fraction *f*_*n*_ of patches across degrees are subject to antimicrobial use. Hence, *ϵ*_*a*,*k*_ = (*p*(*k*)(1 − *f*_*n*_) − *a*_*r*,*k*_ − *a*_*s*,*k*_) and *ϵ*_*n*,*k*_ = (*p*(*k*)*f*_*n*_ − *n*_*r*,*k*_ − *n*_*s*,*k*_), where *p*(*k*) is the probability that a randomly chosen patch in our metapopulation has degree *k*. As with Models (1), (2), Model (3) can represent a regime in which only certain types of sites (e.g., farms and hospitals) receive antimicrobials. Model (3) differs from the previous models in allowing us to further account for the variable connectivities of those sites and sites that may not receive the antimicrobial.

We now specify the migration rates $k\sum_{k^{\prime }}p\left(k^{\prime }|k\right)D_{x,i,k,k^{\prime }}$ of strain *i* into a patch of type *x* of degree *k*. If 〈*k*〉 is the average connectivity of a patch in the metapopulation, and if the average number of links connected to patches with degree *k*′ is *k*′*p*(*k*′), then *p*(*k*′)*k*′/〈*k*〉 describes the probability that a node associated with a randomly chosen link in our metapopulation has degree *k*′.

In the analyses that follow, we assume that the metapopulation can be represented as a random graph with an uncorrelated network. Thus, the proportion *p*(*k*′ | *k*) of links between patches with degrees *k* and *k*′ is independent of *k*. Consequently, the probability that a link associated with a node of degree *k* connects to a node of degree *k*′ is merely the same probability that any given link connects to a node of degree *k*′; hence, *p*(*k*′ | *k*) = *p*(*k*′)*k*′/〈*k*〉 (e.g., Colizza & Vespignani, 2008; Newman et al., 2001).

We model a situation where each of the *k*′ edges receives an equal share of net migrants out of patches of degree *k*′, and the number of migrants of strain *i* is proportional of frequency of patches occupied by strain *i*. Therefore, the weight of any given edge emanating from a patch with degree *k*′ (in effect, a measure of migration intensity) is proportional to $\left(a_{i,k^{\prime }}+n_{i,k^{\prime }}\right)/k^{\prime }$. Finally, we define the quantity *D*(*x*,* y*) to characterizes the ability of strain *y* to establish in a patch of type *x*. Thus, *D*(*x*,* y*) = *c*_*y*_ if *x* is an empty patch, and *D*(*x*,* y*) = *g*_*j*,*y*_ if *x* is a patch already occupied by strain *j*.

Taken together, provided the degree of a patch does not affect the ability of strains that traversed a given link to establish in a new patch, the above considerations imply that the magnitude of migration of strain *i* to a patch of type *x* of degree *k* is therefore

$$\begin{array}{cc} k\sum_{k^{\prime }}p\left(k^{\prime }|k\right)D_{x,i,k,k^{\prime }}= & \frac{k}{<k>}\sum_{k^{\prime }}p\left(k^{\prime }\right)k^{\prime }D\left(x,i\right)\left(a_{i,k^{\prime }}+n_{i,k^{\prime }}\right)/k^{\prime }\\ = & \frac{D(x,i)k}{<k>}\sum_{k^{\prime }}p\left(k^{\prime }\right)\left(a_{i,k^{\prime }}+n_{i,k^{\prime }}\right). \end{array}$$

We note that we can recover an analogous modification of Equations (1), where antimicrobial‐sensitive strains cannot persist in sites where an antimicrobial is used even in the absence of the resistant strain, as a special case of Model (3) with *e*_*a*,*s*_ → ∞. Similarly, when *k* → ∞ and *p*(∞) = 1, Model (3) recovers Model (2).

Finally, we consider whether subjecting a particular type of patch to antimicrobial use promotes the spread of resistant strains. To this end, we consider a metapopulation characterized by a bipartite graph, in which pathogens only migrate between sites with an antimicrobial and sites without an antimicrobial. We highlight two contrasts with the scenario described above, where we vary the number of migration edges across sites and include degree distributions with most patches being minimally connected in our analysis. First, we assume here that each site with the antimicrobial is accessible from each site without it. This might be a reasonable approximation, for example, for an antimicrobial‐resistant pathogen spreading between cities connected through several key transportation hubs where, frequently, links are available to many nonhubs from central nodes, and there is little to no direct connection among the nonhubs (e.g., Nicolaides et al., 2012). Restricting antibiotic use in highly connected cities may be one strategy to halt the spread of antibiotic resistance. We seek to characterize a situation where, for example, the hub cities are not subject to antimicrobial use, but the nonhub cities are. Tolerating the persistence of antimicrobial‐sensitive strains in the former sort of locations has the potential to bottleneck the spread of resistant strains.

Disjoint spatial structures can give rise to distinct sites that also differ in their antimicrobial use. Such scenarios may arise in urban settings where pathogens circulate from one kind of habitat (e.g., residences) to another type of habitat (e.g., clinics and hospitals) back to their original habitat type (e.g., other residences). For instance, households where antimicrobials may not be used may be connected via human movement through common sites, such as hospitals, where antimicrobials are used. However, the effectiveness of such a strategy will depend on several of the same considerations discussed earlier (pleiotropy between antimicrobial resistance and competitive ability, the colonization and local extinction rates of the pathogen, etc.). Such patterns could also emerge, for instance, among certain waterborne pathogens spreading via a water source (where an antimicrobial might be used) to individual homes or communities (where an antimicrobial is not used) rather than directly between individual communities and homes. Foodborne pathogens moving between farms or centralized distribution centers and distal sites (e.g., restaurants or retail establishments) may also exhibit similar patterns of bipartite movement across different types of sites that might be subject to different antimicrobial regimes.

These considerations can be represented as follows. We revise Model (2) so that migration predominantly occurs between patches with or without the antimicrobial, rather than among sites with or without the antimicrobial. Consequently, Equations (2) can be modified as

(4) $$\begin{array}{cc} \frac{dn_{r}}{dt}= & c_{r}a_{r}\left(f_{n}-n_{r}-n_{s}\right)-e_{n,r}n_{r}-g_{r,s}a_{s}n_{r}+g_{s,r}a_{r}n_{s}\\ \frac{da_{r}}{dt}= & c_{r}n_{r}\left(\left(1-f_{n}\right)-a_{r}-a_{s}\right)-e_{a,r}n_{r}+c_{r}a_{s}n_{r}\\ \frac{dn_{s}}{dt}= & c_{s}a_{s}\left(f_{n}-n_{r}-n_{s}\right)-e_{n,s}n_{s}-g_{s,r}a_{r}n_{s}+g_{r,s}a_{s}n_{r}\\ \frac{da_{s}}{dt}= & c_{r}n_{s}\left(\left(1-f_{n}\right)-a_{r}-a_{s}\right)-e_{a,s}s_{r}-c_{r}a_{s}n_{r}. \end{array}$$

Model (4) thus describes a situation where the migrating pathogen must alternate between a patch with the antimicrobial and a patch without the antimicrobial (somewhat akin to the mixing strategy described in Bergstrom et al., 2004). Model (4) characterizes situations where a pathogen must move between two habitat types by allowing antimicrobial use to be targeted to only a certain habitat type—all patches of a single habitat type are subject to antimicrobial use, and all patches of the other habitat type do not receive the antimicrobial. Consequently, *f*_*n*_ describes not only the restricted antimicrobial use in the metapopulation, but also the proportion of patches of a certain type.

In addition to building in spatial heterogeneity in antimicrobial exposure, Model (4) also adds an element of temporal heterogeneity to the pathogen's adaptive landscape. One implication of this is that when *f*_*n*_ is very close to zero or one, our model can also describe situations where certain sites that act as hubs are targeted for antimicrobial use, and where pathogen movement is primarily between hubs and nonhubs. Under these conditions, Model (4) can be interpreted as a variation on Model (3), where most sites have very few edges and a few sites have a large number of edges, and where antimicrobial use varies across the two types of sites. For instance, if *f*_*n*_ is very large, this can describe a situation where a handful of hub sites receive the antimicrobial, whereas the majority nonhub sites do not undergo antimicrobial application. By contrast, when *f*_*n*_ is very small, this can represent a scenario where the majority nonhub sites receive the antimicrobial, whereas the handful of the hub sites do not have the antimicrobial treatment.

### 2.3 Model analyses and results

We use analytical and numerical methods to evaluate how varying the spatial prevalence (1 − *f*_*n*_) of antimicrobial use affects the dynamics of antimicrobial resistance. We focus on two key questions regarding the interplay between pleiotropic fitness costs and the extent of antimicrobial use over space: (a) How might restricting the spatial extent of antimicrobial use interact with the pleiotropic costs and the baseline migration rate to prevent the emergence of antimicrobial resistance in the metapopulation? and (b) Provided resistance has emerged, how does varying the spatial use of antimicrobials, together with the pleiotropic effect of resistance on colonization and competition, affect both the prevalence of antimicrobial‐resistant strains and the pathogen itself?

Several pathogens have been shown to be able to circumvent the fitness costs associated with evolved resistance (e.g., Marcusson, Frimodt‐Møller, & Hughes, 2009; Schrag, Perrot, & Levin, 1997). In particular, compensatory mutations and coselection for fitness‐enhancing alleles can improve the competitive performance of resistant strains, viz., antimicrobial‐sensitive strains (e.g., Handel, Regoes, & Antia, 2006; Melnyk et al., 2015; Schrag & Perrot, 1996). Thus, we also assess throughout our analyses how the strength of the trade‐off between antimicrobial resistance and competitive performance mediates whether varying antimicrobial use over space reduces the risk of resistance emerging.

#### 2.3.1 How does the interplay between pleiotropic effects on colonization and competition interact with the spatial extent of antimicrobial use to affect the emergence of antimicrobial resistance in the metapopulation?

##### 2.3.1.1 The antimicrobial‐sensitive pathogen cannot establish in sites with the antimicrobial

We assess the emergence of antimicrobial resistance in a metapopulation by analyzing Models (1), (2), (3), (4) to identify the conditions under which a resistant strain can invade when the antimicrobial‐sensitive strain is at equilibrium. Thus, if a resistant strain is able to spread beyond a single patch, we consider the strain as being emergent in the metapopulation.

We begin with Model (1), in which the antimicrobial‐sensitive strain cannot occupy patches in which the antimicrobial is present, and assume the ancestral pathogen is susceptible to the antimicrobial. In the absence of antimicrobial resistance, the equilibrium prevalence of the pathogen across sites is $n_{s}^{*}=f_{n}-e_{n,s}/c_{s}$. This equilibrium is viable provided the colonization rate of the pathogen exceeds the local extinction rate scaled by the fraction of patches in which the antimicrobial is absent (i.e., *c*_*s*_ > *e*_*n*,*s*_/*f*_*n*_). Hence, increasing the prevalence of antimicrobial use toward one rapidly reduces the equilibrium prevalence of the pathogen before antimicrobial resistance evolves.

Provided the antimicrobial‐sensitive pathogen is at equilibrium and this equilibrium is viable, antimicrobial resistance can be prevented from emerging in Equations (1) when *e*_*a*,*r*_ exceeds (*c*_*r*_(*c*_*s*_ + *e*_*n*,*s*_ − *c*_*s*_ *f*_*n*_) + *e*_*n*,*s*_(*g*_*r*,*s*_ − *g*_*s*,*r*_))/*c*_*s*_ − *c*_*s*_(*e*_*n*,*r*_ + *f*_*n*_(*g*_*r*,*s*_ − *g*_*s*,*r*_)) and

(5) $$\begin{array}{cc} g_{s,r}-g_{r,s} & <\frac{c_{s}e_{n,r}}{c_{s}f_{n}-e_{n,s}}\;\text{and}\\ c_{r}< & \frac{e_{a,r}\left(c_{s}\left(e_{n,r}+f_{n}\left(g_{r,s}-g_{s,r}\right)\right)+e_{n,s}\left(g_{s,r}-g_{r,s}\right)\right)}{e_{n,s}\left(e_{a,r}+\left(f_{n}-1\right)g_{r,s}\right)-c_{s}\left(f_{n}-1\right)\left(e_{n,r}+f_{n}g_{r,s}\right)}. \end{array}$$

(see Supporting Information Appendix S1 for details). We highlight some implications of Conditions (5). First, even if antimicrobial use is minimal (*f*_*n*_ large), preventing the emergence of antimicrobial resistance still requires the competitive asymmetry between strains in sites without the antimicrobial (*g*_*s*,*r*_ − *g*_*r*,*s*_) to be less than the extinction rate of the resistant strain in sites without the antimicrobial, scaled by the equilibrium fraction of pathogen‐occupied patches. Because the equilibrium for pathogen‐occupied patches is larger if fewer sites receive the antimicrobial, the importance of the competitive asymmetry to preventing the emergence of antimicrobial resistance increases as fewer sites use the antimicrobial and decreases as more sites adopt the antimicrobial.

Second, suppressing the emergence of antimicrobial resistance still requires the ability *c*_*r*_ of the resistant strain to colonize new sites to not exceed $\frac{e_{a,r}\left(c_{s}\left(e_{n,r}+f_{n}\left(g_{r,s}-g_{s,r}\right)\right)+e_{n,s}\left(g_{s,r}-g_{r,s}\right)\right)}{e_{n,s}\left(e_{a,r}+\left(f_{n}-1\right)g_{r,s}\right)-c_{s}\left(f_{n}-1\right)\left(e_{n,r}+f_{n}g_{r,s}\right)}$. This illustrates a fundamental tension between preventing antimicrobial resistance at a global scale and eradicating the pathogen locally. If antimicrobial use is rare (so that *f*_*n*_ large), the evolution of antimicrobial resistance can be mitigated by encouraging local persistence of the antimicrobial‐sensitive pathogen. For example, if the colonization rate of the resistant pathogen can be determined, reducing the antimicrobial‐sensitive strain's per‐patch extinction rate *e*_*n*,*s*_ or increasing its colonization rate *c*_*s*_ in response across the landscape can prevent the emergence of antimicrobial resistance. By contrast, if antimicrobial use is very common (so that *f*_*n*_ is small), then the second inequality in Conditions (5) implies that no amount of local persistence of the antimicrobial‐sensitive strain can prevent the resistant strain from invading as long as the resistant strain's colonization rate exceeds its extinction rate in patches with the antimicrobial.

**Figure 1:**
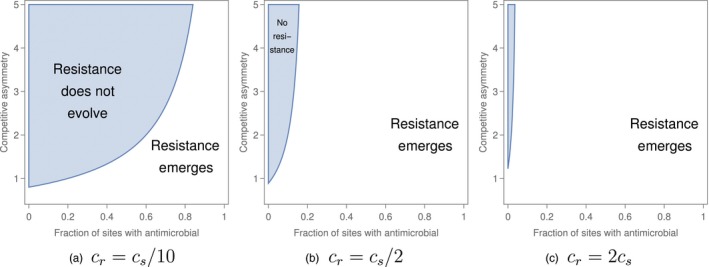
The ability of an antimicrobial strain to emerge in a metapopulation where the ancestral, antimicrobial‐sensitive strain is unable to colonize patches where an antimicrobial is used. Here, and in Figures 2 and 3, emergent resistance (unshaded regions) is illustrated as a function of the fraction of patches receiving the antimicrobial (horizontal axis) and the strength of the competition–resistance trade‐off (vertical axis). (a) The ability of the resistant strain to colonize empty patches is one‐tenth the ability of the antimicrobial‐sensitive strain to colonize empty patches, (b) the colonization ability of the resistant strain is one half the colonization ability of the antimicrobial‐sensitive strain, and (c) the colonization ability of the resistant strain is twice the colonization ability of the antimicrobial‐sensitive strain. In this, and in subsequent figures, the competitive asymmetry is defined as the ability *g*_*r*,*s*_ of the antimicrobial‐sensitive strain to displace the resistant strain in a given patch, relative to the ability *g*_*s*,*r*_ of the resistant strain to displace the antimicrobial‐sensitive strain in a patch. Other parameter values are *c*_*s*_ = 0.1, *e*_*n*,*s*_ = *e*_*n*,*r*_ = *e*_*a*,*r*_ = 0.01 and *g*_*s*,*r*_ = 0.05. For more on the numerical parameter ranges, see the section “How does varying the spatial extent of antimicrobial use affect the prevalence of antimicrobial resistance across the landscape?” in the main text

Figure 1 illustrates how reducing the fraction of patches in which the antimicrobial is applied can prevent the emergence of resistance across different levels of the competitive asymmetry (i.e., the ratio of the ability *g*_*r*,*s*_ of the antimicrobial‐sensitive strain to competitively displace the resistant strain to the ability *g*_*s*,*r*_ of the antimicrobial‐resistant strain to competitively displace the sensitive strain) and the migration rate of the resistant strain. When the resistant strain is a poor migrant relative to the antimicrobial‐sensitive strain, reducing the fraction of patches in which the antimicrobial is used keeps the pathogen population susceptible to the antimicrobial, provided the competitive asymmetry between the strains is large and favors the antimicrobial‐sensitive strain in sites without the antimicrobial. If the competitive asymmetry between the two strains is modest, the emergence of antimicrobial resistance can still be prevented if the fraction of sites using the antimicrobial is also modest.

##### 2.3.1.2 The antimicrobial‐sensitive pathogen can establish in sites in the presence of the antimicrobial

Next, we analyze invasion of the resistant strain in Model (2) in which the antimicrobial‐sensitive pathogen can still occupy antimicrobial sites, and all sites are equally accessible from any other site.

In Model (2), in the absence of antimicrobial resistance, the equilibrium patches occupied by the antimicrobial‐sensitive strain are given by:

(6) $$\begin{array}{cc} a_{s}^{*}= & \frac{c_{s}\left(-2e_{a,s}f_{n}+e_{a,s}+2e_{n,s}\left(f_{n}-1\right)\right)+e_{a,s}\left(\sqrt{4c_{s}f_{n}\left(e_{a,s}-e_{n,s}\right)+{\left(c_{s}-e_{a,s}+e_{n,s}\right)}^{2}}-e_{a,s}+e_{n,s}\right)}{2c_{s}\left(e_{a,s}-e_{n,s}\right)}\\ & \text{and}\\ n_{s}^{*}= & -\frac{e_{n,s}\left(\sqrt{4c_{s}f_{n}\left(e_{a,s}-e_{n,s}\right)+{\left(c_{s}-e_{a,s}+e_{n,s}\right)}^{2}}+e_{a,s}-e_{n,s}\right)-c_{s}\left(2e_{a,s}f_{n}-2e_{n,s}f_{n}+e_{n,s}\right)}{2c_{s}\left(e_{a,s}-e_{n,s}\right)}. \end{array}$$

If the fraction *f*_*n*_ of sites without the antimicrobial is too small to permit persistence in the absence of the ability to establish in antimicrobial sites (so that *f*_*n*_ < *e*_*n*,*s*_/*c*_*s*_; see the results for Model (1)), then equilibrium (6) is viable only if *e*_*n*,*s*_ < *e*_*a*,*s*_ < (*c*_*s*_ *e*_*n*,*s*_(*f*_*n*_ − 1))/(*c*_*s*_ *f*_*n*_ − *e*_*n*,*s*_). We highlight how this illustrates the contribution of the extinction rate in patches with the antimicrobial to the pathogen's viability in the landscape. In particular, as the proportion of sites with antimicrobials increases, the viability condition reduces to the requirement that the extinction rate of the antimicrobial‐sensitive strain in patches with the antimicrobial cannot exceed the colonization rate of the antimicrobial‐sensitive strain.

Although Model (2) exhibits strong nonlinearities, we show in Supporting Information Appendix S2 that if the extinction rate *e*_*a*,*r*_ of the resistant strain in sites with the antimicrobial is less than $c_{r}-e_{n,r}-g_{r,s}a_{s}^{*}-\left(c_{r}+g_{r,s}-g_{s,r}\right)n_{s}^{*}$ (where $n_{s}^{*}$ and $a_{s}^{*}$ are as in Equations (6)), then the resistant strain can invade. If, however, the extinction rate of the resistant strain exceeds this threshold, a resistant strain can be prevented from emerging provided

(7) $$\begin{array}{cc} f_{n}<\frac{e_{a,r}\left(e_{n,r}+a_{s}^{*}g_{r,s}+\left(g_{r,s}-g_{s,r}\right)n_{s}^{*}\right)+c_{r}\left(e_{n,r}-e_{a,r}n_{s}^{*}+g_{r,s}\left(a_{s}^{*}+n_{s}^{*}\right)\right)}{c_{r}\left(e_{n,r}+g_{r,s}\left(a_{s}^{*}+n_{s}^{*}\right)-e_{a,r}\right)}\text{and} & \\ g_{s,r}<\frac{e_{n,r}+g_{r,s}\left(a_{s}^{*}+n_{s}^{*}\right)}{n_{s}^{*}}. \end{array}$$

A key implication of this result is that if there is substantial competitive asymmetry between the strains (i.e., *g*_*r*,*s*_ is very large relative to *g*_*s*,*r*_), the resistant strain cannot invade so long as the fraction of sites without the antimicrobial does not exceed the sum of the resistant strain's colonization and extinction rate scaled by the resistant strain's colonization rate (see Supporting Information Appendix S2). Furthermore, if most sites are exposed to the antimicrobial and the equilibrium fraction of sites without the antimicrobial occupied by the antimicrobial‐sensitive strain is small (so that $f_{n},n_{s}^{*}\to 0$), then Conditions (7) imply that whenever the colonization ability of resistant strains is less than their extinction rate in sites with the antimicrobial, then antimicrobial resistance cannot emerge.

**Figure 2:**
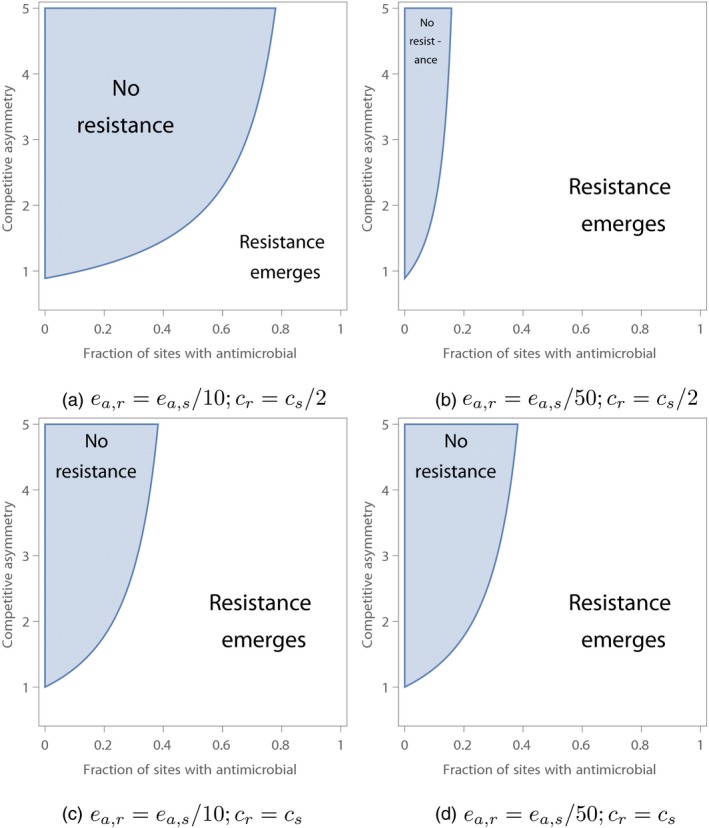
The ability of an antimicrobial strain to emerge in a metapopulation where the ancestral, antimicrobial‐sensitive strain can colonize patches where an antimicrobial is used. As in Figure 1, resistance fails to emerge in the shaded region and emerges in the unshaded region. (a) The resistant strain has an extinction rate in sites with the antimicrobial 10× smaller than the antimicrobial‐sensitive strain's extinction rate in sites with the antimicrobial, and the ability of the resistant strain to colonize empty patches is half the colonization ability of the antimicrobial‐sensitive strain. (b) The resistant strain has an extinction rate in sites with the antimicrobial 50× smaller than the antimicrobial‐sensitive strain's extinction rate in sites with the antimicrobial, and the ability of the resistant strain to colonize empty patches is half the colonization ability of the antimicrobial‐sensitive strain. (c) The resistant strain has an extinction rate in sites with the antimicrobial 10× smaller than the antimicrobial‐sensitive strain's extinction rate in sites with the antimicrobial, and the ability of the resistant strain to colonize empty patches is equal to the colonization ability of the antimicrobial‐sensitive strain. (d) The resistant strain has an extinction rate in sites with the antimicrobial 50× smaller than the antimicrobial‐sensitive strain's extinction rate in sites with the antimicrobial, and the ability of the resistant strain to colonize empty patches is equal to the colonization ability of the antimicrobial‐sensitive strain. In all panels, *e*_*a*,*s*_ = 50*e*_*n*,*s*_; reducing the extinction rate *e*_*a*,*s*_ of the antimicrobial‐sensitive strain in sites with the antimicrobial had little effect (results not shown). All other parameter values are as in Figure 1

The combined effects of restricting the spatial extent of antimicrobial use and varying the competitive asymmetry of the two strains are broadly similar to the case where the antimicrobial‐sensitive pathogen could not colonize sites with the antimicrobial (Figure 2). The ability of the resistant strain to avoid extinction in patches with the antimicrobial alters the region of parameter space in which resistance can emerge (Figure 2). When the extinction rate *e*_*a*,*r*_ of the resistant strain in patches where the antimicrobial is present is greater than the extinction rate *e*_*n*,*s*_ of the antimicrobial‐sensitive strain in patches without the antimicrobial, then the emergence of antimicrobial resistance can be prevented even when antimicrobial use is common, provided the competitive asymmetry is sufficiently strong (Figure 2a). By contrast, if the extinction rate of the resistant strain in sites with the antimicrobial is comparable to the extinction rate of the antimicrobial‐sensitive strain in sites without the antimicrobial (Figure 2b), then resistance emerges much more readily. Increasing the colonization rate of the resistant strain serves to homogenize the landscape, thereby mitigating this effect (e.g., Figure 2a vs. 2b and Figure 2c vs. 2d).

##### 2.3.1.3 Patches exhibit differential connectivities

The high dimensionality of Model (3) renders the boundary equilibria, much less the invasion criteria of an antimicrobial‐resistant strain in a metapopulation consisting of only antimicrobial‐sensitive pathogens, algebraically intractable. Thus, we defer exploring the behavior of Model (3) to our numerical analyses later, where we will compare the long‐term behavior of antimicrobial resistance spread across alternative metapopulation connectivities in greater detail.

Instead, here we highlight analytic results for the limiting case in which patches that are highly connected either receive the antimicrobial or do not (i.e., Model 4 when *f*_*n*_ ≈ 1 or *f*_*n*_ ≈ 0), and the pathogen must alternately migrate between sites with and without the antimicrobial. The former situation can arise, for instance, with foodborne pathogens where the antimicrobial is applied at, for example, distribution centers, but is not used at other sites, such as individual restaurants or retail establishments. We ask how the targeted use of antimicrobials either at such sites can create bottlenecks to pathogen migration which can, in turn, affect the emergence of antimicrobial resistance.

When only the antimicrobial‐sensitive strain is present in the metapopulation, the interior equilibrium of Model (4) is given by

(8) $$\begin{array}{cc} n_{s}^{*}= & \frac{e_{a,s}e_{n,s}+c_{s}^{2}\left(f_{n}-1\right)f_{n}}{c_{s}^{2}f_{n}-c_{s}\left(c_{s}+e_{n,s}\right)}\text{and}\\ a_{s}^{*}= & \frac{c_{s}^{2}\left(1-f_{n}\right)f_{n}-e_{a,s}e_{n,s}}{c_{s}\left(e_{a,s}+c_{s}f_{n}\right)}. \end{array}$$

This equilibrium is viable whenever $e_{n,s}<c_{s}^{2}f_{n}\left(1-f_{n}\right)/e_{a,s}$. Thus, an antimicrobial‐sensitive strain highly sensitive to the antimicrobial (*e*_*a*,*s*_ → ∞) must have a correspondingly strong ability to persist in sites without the antimicrobial. We also note that this condition governs the stability of the pathogen‐free equilibrium in the absence of the resistant strain, with the pathogen‐free equilibrium always unstable whenever the boundary equilibrium consisting of the antimicrobial‐sensitive strain is viable.

We show in Supporting Information Appendix S3 that a resistant strain can be prevented from invading system (4) if and only if

(9) $$\begin{array}{c} \frac{{\left(e_{a,r}-a_{s}^{*}g_{r,s}-e_{n,r}\right)}^{2}-4c_{r}^{2}\left(1-f_{n}\right)\left(f_{n}-n_{s}^{*}\right)}{4c_{r}\left(1-f_{n}\right)n_{s}^{*}}\leq g_{s,r}<\frac{e_{a,r}\left(a_{s}^{*}g_{r,s}+e_{n,r}\right)-c_{r}^{2}\left(1-f_{n}\right)\left(f_{n}-n_{s}^{*}\right)}{c_{r}\left(1-f_{n}\right)n_{s}^{*}}. \end{array}$$

This result suggests a role for the trade‐off between competition and resistance in governing the emergence of antimicrobial resistance when the pathogen must alternate between sites with and without the antimicrobial. In particular, as the resistant strain becomes increasingly capable of persisting for very long periods of time in sites with the antimicrobial (so that *e*_*a*,*r*_ → 0), the ability *g*_*r*,*s*_ of the antimicrobial‐sensitive strain to displace the resistant strain has less effect on the ability to prevent the emergence of resistance. This is because if the resistant strain is able to persist indefinitely in sites with the antimicrobial, as a larger proportion of sites receive the antimicrobial, the evolutionary viability of the antimicrobial resistance strain reduces to the requirement that the competitive displacement rate *g*_*s*,*r*_ of the antimicrobial‐sensitive strain by the resistant strain exceed *c*_*r*_. Thus, as more sites receive the antimicrobial, if the antimicrobial‐resistant strain is less able to displace the susceptible strain (*g*_*s*,*r*_ small), sites without the antimicrobial can serve as bottlenecks preventing the spatial spread of the resistant strain.

**Figure 3:**
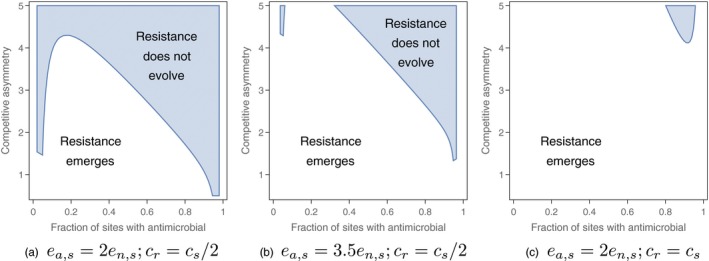
The ability of a resistant strain to emerge in a metapopulation where migration can only happen between patches with and without the antimicrobial. As in Figures 1 and 2, resistance fails to emerge in the shaded region and emerges in the unshaded region. When few patches receive the antimicrobial (1 − *f*_*n*_ small), patches receiving the antimicrobial act as hubs, whereas when most patches receive the antimicrobial (1 − *f*_*n*_ large), the patches without the antimicrobial act as migratory hubs. (a) The antimicrobial‐sensitive strain goes extinct twice as quickly in patches with the antimicrobial compared to patches without the antimicrobial, and the colonization rate of the resistant strain in empty patches is half the colonization rate of the antimicrobial‐sensitive strain in empty patches. (b) The extinction rate of the antimicrobial‐sensitive strain is 3.5 X larger in sites with the antimicrobial than in sites without the antimicrobial, and the colonization rate of the resistant strain is half the colonization rate of the antimicrobial‐sensitive strain. (c) The antimicrobial‐sensitive strain goes extinct twice as quickly in patches with the antimicrobial compared to patches without the antimicrobial, and the resistant strain's colonization rate is the same as that of the antimicrobial‐sensitive strain. Other parameter values are as in Figure 1

Therefore, in contrast to the models without pathogens alternating between sites with and without the antimicrobial, we find that when the competitive differential between strains is substantial (i.e., *g*_*r*,*s*_/*g*_*s*,*r*_ large), the emergence of antimicrobial resistance can be prevented, particularly when antimicrobial use is common (Figure 3). When only a few patches are exempt from receiving the antimicrobial, even a smaller competitive asymmetry in sites without the antimicrobial can suffice to prevent the emergence of resistance in the presence of such spatial bottlenecks between sites where the antimicrobial is present (Figure 3a). Our results suggest that under some conditions, even when antimicrobial sensitivity cannot confer substantial competitive advantages, focusing antimicrobial treatment on the majority of sites (*f*_*n*_ → 0) can potentially prevent the emergence of antimicrobial resistance. We note, however, that prospects for relying on sites without the antimicrobial diminish as the per‐site migration rate of the resistant strain increases, or as the extinction rate of the antimicrobial‐sensitive strain increases (Figure 3b,c).

#### 2.3.2 How does varying the spatial extent of antimicrobial use interact with the pleiotropic effects on colonization and competition to affect the prevalence of antimicrobial resistance across the landscape?

Whereas ensuring the long‐term efficacy of antimicrobial drugs to treat infections requires, in part, preventing the emergence of antimicrobial resistance in the first place, understanding the long‐term dynamics of antimicrobial resistance in spatially structured environments where antimicrobial resistance has already emerged can also help guide management regimes. If the emergence of antimicrobial resistance cannot be prevented altogether, then mitigating the spatial spread of antimicrobial resistance becomes important. In particular, understanding the long‐term dynamics of antimicrobial resistance is critical to evaluating whether the emergence of antimicrobial resistance permits the pathogen to expand into previously unoccupied patches. Therefore, we numerically integrated Models (1), (2), (3), (4) to assess their long‐term behavior. For simplicity and tractability, we assume all intervention strategies begin only when the antimicrobial‐sensitive strain is present in systems (1–4). In the case of (3), we initialized the fraction of occupied sites of a particular degree by weighing the degree distribution by the fraction of antimicrobial‐sensitive strains at equilibrium in Model (2). We focus our numerical analyses on two key parameters: the extent of antimicrobial use across space (varied from none to complete) and the competitive asymmetry (varied across a factor of 10, from the resistant strain being five times as competitively effective as the antimicrobial‐sensitive strain, to being only a fifth as competitively effective as the antimicrobial‐sensitive strain). In practice, feasible management regimes may occupy much narrower regions of parameter space. By presenting a deliberately wide range of parameter space, we hope to convey a heuristic impression of how different parameter combinations alter the spatial spread of antimicrobial resistance. Furthermore, we present the effect of varying the baseline colonization and extinction rates. In particular, to evaluate whether the relative colonization and extinction rates of the two strains affect our qualitative predictions, we simulated our models across four qualitatively distinct situations: (a) where the resistant strain is half as effective at colonization than the antimicrobial‐sensitive strain and the antimicrobial‐sensitive strain has a 50‐fold higher extinction rate in sites with the antimicrobial, (b) where the resistant strain is considerably more effective at colonization than the antimicrobial‐sensitive strain and the antimicrobial‐sensitive strain has a 50‐fold higher extinction rate in sites with the antimicrobial, (c) where the resistant strain is half as effective at colonization than the antimicrobial‐sensitive strain but the antimicrobial‐sensitive strain is only twice as likely to suffer extinction in sites with the antimicrobial than without, and (d) where the resistant strain is considerably more effective at colonization than the antimicrobial‐sensitive strain and the antimicrobial‐sensitive strain is only twice as likely to suffer extinction in sites with the antimicrobial than without. Numerical analyses were carried out using the deSolve package in R (Soetaert, Petzoldt, & Setzer, 2010).

**Figure 4:**
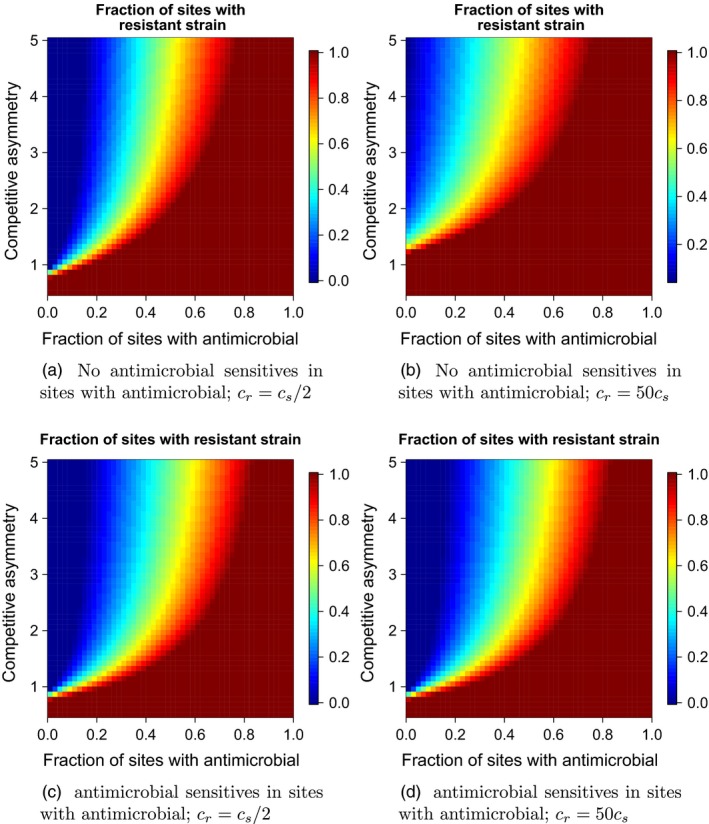
The long‐term equilibrium frequency of patches with a resistant strain as a function of the fraction of patches where the antimicrobial is used and the relative competitive asymmetry between the resistant and antimicrobial‐sensitive strain. (a) Antimicrobial‐sensitive pathogens cannot occupy sites where the antimicrobial is present, and the colonization ability of the resistant strain is half the colonization ability of the antimicrobial‐sensitive strain. (b) Antimicrobial‐sensitive pathogens cannot occupy sites where the antimicrobial is present, and the colonization ability of the resistant strain is 50× the colonization ability of the antimicrobial‐sensitive strain. (c) Antimicrobial‐sensitive pathogens cannot occupy sites where the antimicrobial is present, and the colonization ability of the resistant strain is half the colonization ability of the antimicrobial‐sensitive strain. (d) Antimicrobial‐sensitive pathogens cannot occupy sites where the antimicrobial is present, and the colonization ability of the resistant strain is 50× the colonization ability of the antimicrobial‐sensitive strain. In panels (c, d), *e*_*a*,*s*_ = 2*e*_*n*,*s*_; note as *e*_*a*,*s*_ → ∞, Model (2) approaches Model (1). All other parameter values are as in Figure 1

Figure 4 illustrates how the extent of spatial antimicrobial use and the competition–resistance trade‐off govern the long‐term dynamics of Models (1), (2). When the pathogen can migrate between any sites, a resistant strain can dominate the entire landscape when a large majority of patches are subject to antimicrobial use, even when the trade‐off between competitive ability and resistance is strong (Figure 4). As was the case with our analyses of the emergence of antimicrobial resistance, we find that the competition–resistance trade‐off is only effective at preventing the spatial dominance of antimicrobial resistance when relatively few sites are exposed to the antimicrobial. Nevertheless, when the competition–resistance trade‐off is weak (e.g., when the resistant strain can readily evolve compensatory mutations), then no amount of spatial restrictions on antimicrobial use can prevent eventual replacement of the antimicrobial‐sensitive strain. Limiting migration of the resistant strain can have a modest effect on preventing the spatial spread antimicrobial resistance; restricting the spatial scale of antimicrobial use is most effective when the migration rate of the resistant strain is limited (Figure 4a and 4c vs. Figure 4b and 4d).

The results above are based on Models (1), (2) that permit pathogens to move from any single patch to any other patch. Our results sofar thereby provide a set of baseline expectations for the long‐term spatial prevalence of antimicrobial resistance in the metapopulation. In the analyses that follow, we compare these results to Models (3), (4) in which patches vary in their connectivity.

**Figure 5:**
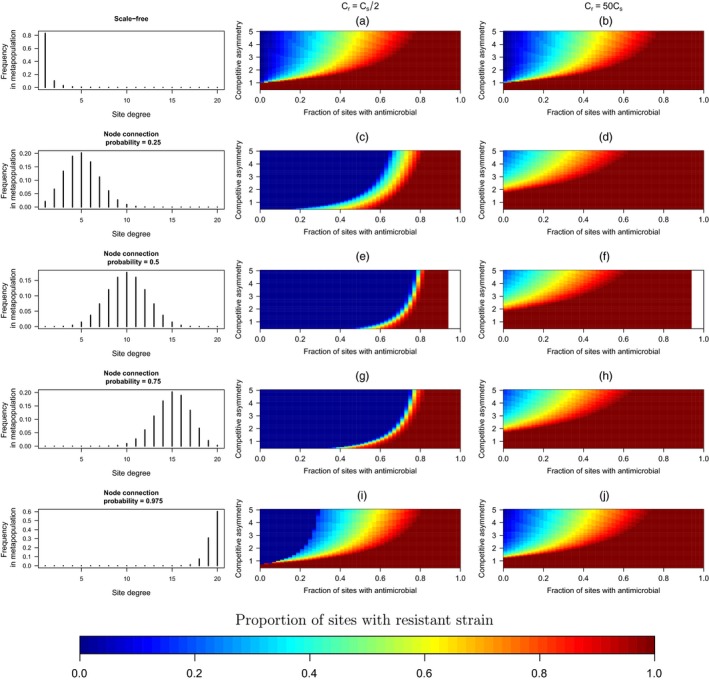
The long‐term equilibrium frequency of patches with a resistant strain as a function of the fraction of patches where antimicrobial use is constrained, the relative competitive asymmetry and colonization rates between the resistant and antimicrobial‐sensitive strain, and the distribution of connectivities among sites (the degree distribution). The various degree distributions are illustrated in the first column. Degree distributions used in the analyses are (a, b) a scale‐free degree distribution (*P*(*k*) ∼ *k* ^−3^), and Bernoulli random networks with node connection probabilities of (c, d) 0.25, (e, f) 0.5, (g, h) 0.75, and (i, j) 0.975

For Model (3), we present results from five qualitatively distinct degree distributions along a continuum from metapopulations where most patches are connected to very few patches to metapopulations where most patches exhibit high degrees of connectivities. Across degree distributions, as with Models (1), (2) with free migration between patches (the island models), competitive asymmetry can reduce the spread of resistance through the metapopulation when antimicrobial use is constrained (Figure 5). Furthermore, increasing the colonization rate of the resistant strain again reduces the effectiveness of constraining antimicrobial use, particularly when the competitive asymmetry between strains is limited (Figure 5, second column vs. third column panels).

Figure 5 highlights how the effectiveness of constraining antimicrobial use across space depends on the variance of the degree distribution. When the resistant strain's colonization ability is limited, the model predicts both minimally connected and highly connected metapopulations, and where the variance of the degree distribution is low, will exhibit somewhat similar long‐term resistance profiles as functions of constrained antimicrobial use and fitness costs (Figure 5a,i). By contrast, when connectedness varies more among patches, constraining antimicrobial use has considerable potential in preventing antimicrobial resistance from spreading across the metapopulation, even when the competitive fitness costs are minimal. This result, however, does not hold if resistance confers greater migratory potential. Indeed, the effect of the resistant strain's colonization rate on the long‐term, metapopulation‐wide resistance frequency is more pronounced for highly unequal degree distributions (panels c–h) than when most patches are connected to only one other patch (Figure 5a,b), or when there is a high degree of connectivity among patches, as in Figure 5i,j or in the island models (Figure 5 vs. 4). We highlight a key result: In metapopulations with considerable variability in connectedness, constraining antimicrobial use across space only works if the migration of the resistant pathogen is modest; yet the situation is reversed if the resistant strain has a high colonization rate: A highly mobile resistant strain can actually spread more readily in a metapopulation with highly variable patch connectivity.

**Figure 6:**
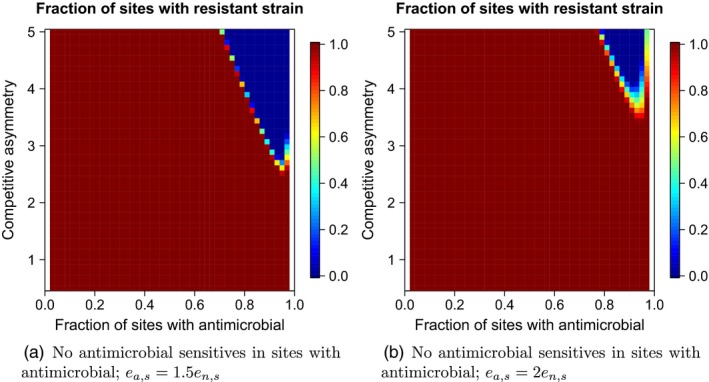
The long‐term fraction of patches occupied by the resistant strain in a metapopulation where migration can only happen between patches with and without the antimicrobial. When few patches receive the antimicrobial (1 − *f*_*n*_ small), those patches act as hubs, whereas when most patches receive the antimicrobial (1 − *f*_*n*_ large), the patches without the antimicrobial act as migratory hubs. (a) The extinction rate of the antimicrobial‐sensitive strain is the same in patches with and without the antimicrobial, and (b) the extinction rate of the antimicrobial‐sensitive strain is two orders of magnitude larger in sites with the antimicrobial. For both panels, *c*_*r*_ = *c*_*s*_; other parameter values are as in Figure 1

Although the analyses above illustrate how patterns of connectivity within a metapopulation can alter antimicrobial resistance emergence across the metapopulation, the basic qualitative result is reminiscent of the case where every patch is connected to every other patch: Constraining antimicrobial use across space can be effective, particularly when there is a strong trade‐off between resistance and competition and limited migration of the resistant strain. By contrast, when the pathogen migrates between patches with and without the antimicrobial (Model 4), a resistant strain that does not experience a strong competitive trade‐off is readily able to establish itself throughout the landscape regardless of the spatial restriction in antimicrobial use (Figure 6). However, as with the invasion analysis, a severe competition–resistance trade‐off can prevent replacement of the antimicrobial‐sensitive strain if the small fraction of sites that do not receive the antimicrobial can act as hubs (i.e., most sites receive the antimicrobial). This effect is most apparent when the antimicrobial‐sensitive strain exhibits a modest ability *e*_*a*,*s*_ to persist in sites with the antimicrobial (Figure 6a vs. 6b).

Finally, one consequence of the spread of antimicrobial resistance through the landscape can be the emergence of an eco‐evolutionary interaction in which the evolution of resistance alters the pathogen's metapopulation dynamics (Post & Palkovacs, 2009). Of particular interest is the case where the evolution of resistance permits the pathogen to colonize a larger share of previously inaccessible patches.

**Figure 7:**
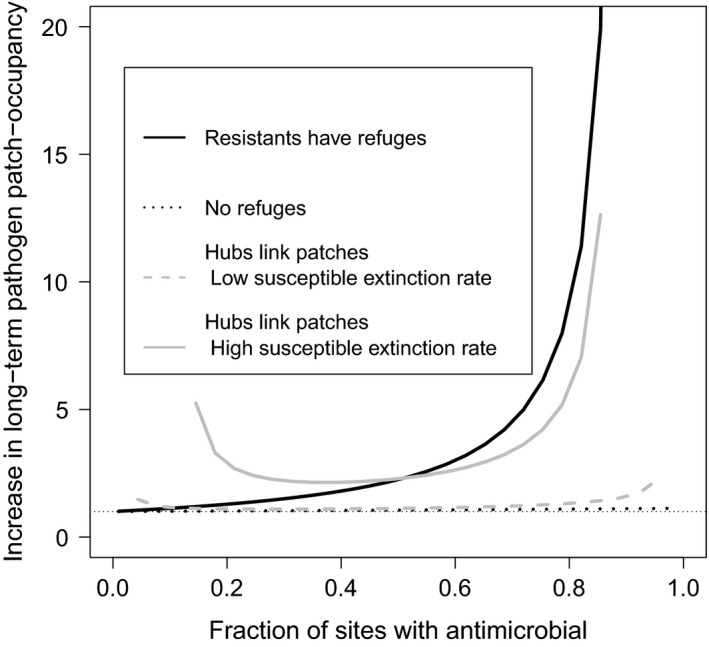
The outcome of an eco‐evolutionary interaction between the evolution of antimicrobial resistance and the pathogen's metapopulation dynamics. The vertical axis represents the factor by which the equilibrium patches occupied across the landscape increases in response to the emergence of antimicrobial resistance. For Model (2), (3), (4), the antimicrobial‐sensitive strain's extinction rate *e*_*a*,*s*_ in patches with the antimicrobial was varied from *e*_*a*,*s*_ = 2*e*_*n*,*s*_ to *e*_*a*,*s*_ = 10*e*_*n*,*s*_; the plotted values are for the cases where *g*_*s*,*r*_ = *g*_*r*,*s*_. All other parameter values are as in Figure 1. We note that when the pathogen can only migrate between sites with the antimicrobial and sites without the antimicrobial, when the antimicrobial‐sensitive strain's extinction rate in sites with the antimicrobial is high, then the ancestral, antimicrobial‐sensitive pathogen cannot persist when too few or too many sites are exposed to the antimicrobial

When the antimicrobial‐sensitive strain is completely unable to establish in sites where the antimicrobial is present, the eco‐evolutionary effect of the emergence of antimicrobial resistance is most apparent when the antimicrobial‐sensitive‐only boundary equilibria are well below the proportion of potentially occupied patches (Figure 7, solid black lines). When the pathogen must alternate between sites, if only a few sites receive the antimicrobial (*f*_*n*_ is large), the boundary, antimicrobial‐sensitive‐only equilibrium can be unstable and this equilibrium is undefined. When this equilibrium is viable, the eco‐evolutionary response following the establishment of antimicrobial resistance exhibits a fundamental asymmetry as more patches receive the antimicrobial (Figure 7, solid gray line). The strong eco‐evolutionary effect is apparent when most patches receive the antimicrobial, and thus, the resistant pathogen becomes able to access a large fraction of hitherto unoccupied patches. However, an eco‐evolutionary interaction is less pronounced when only a handful of patches receive the antimicrobial. This is because in the context of Model (4), such patches can act as hubs. Thus, although the evolution of antimicrobial resistance permits the pathogen to cross such bottlenecks, antimicrobial resistance is advantageous in relatively fewer sites. The net effect of these two processes results in an asymmetric eco‐evolutionary response across a gradient of spatial prevalence in antimicrobial use. However, when the antimicrobial‐sensitive strain is able to better persist in sites with the antimicrobial (gray dashed line), then the asymmetric eco‐evolutionary response is muted as more sites are able to host a pathogen population prior to the emergence of antimicrobial resistance. We add that these results are not affected by increasing the severity of the competition–resistance trade‐off (Supporting Information Figure S1).

## 3 DISCUSSION

Here, we have analyzed a series of metapopulation models describing the emergence and spread of antimicrobial resistance in a pathogen across a landscape. We derived conditions for how the spatial extent of antimicrobial use governs the ability of resistance to emerge under four scenarios: (a) The antimicrobial‐sensitive strain cannot occupy sites where the antimicrobial is in use, (b) the antimicrobial‐sensitive strain can occupy all sites, (c) patches exhibit differential connectivity, and (d) individual pathogens of either strain can only migrate between sites that differ in their antimicrobial use (as could happen, for instance, when antimicrobial use is restricted to certain centralized geographic hubs). We assessed how each of these scenarios changes how the spatial targeting of antimicrobial use governs the evolution of resistance, and illustrated how the competitive ability of the strains in sites without the antimicrobial can modify the dynamics of resistance across space.

As noted in the model descriptions, reinterpreting patches as individual hosts allows one to recover a classical epidemiological model from Equations (2), (3), (4), (5), (6), (7), (8), (9). This suggests that controlling antimicrobial spread in a metapopulation may be more akin to controlling prophylactic‐resistant pathogens in a well‐mixed population than controlling nonprophylactic drug resistance in single populations. Consequently, here we highlight contrasts of the scope of our analyses of Models (2), (3), (4), (5), (6), (7), (8), (9) from select analyses from previous studies exploring prophylaxis resistance in well‐mixed populations (Lipsitch, 1997; McLean, 1995; Park et al., 2015).

Among the models of single host–pathogen communities, that of Park et al. (2015) is perhaps among the most conceptually related to Models ((2), (3), (4), (5), (6), (7), (8), (9) and (4)). Park et al. (2015) show that reducing the extent of prophylactic coverage can create refuges for antimicrobial‐sensitive strains, provided there is a high degree of transmission between treated and untreated hosts. A key distinction between Models (2), (3), (4), (5), (6), (7), (8), (9) and Park et al. (2015) is that our model characterizes the fitness cost as the ease with which superinfection occurs among strains, whereas Park et al. (2015) do not explicitly model superinfection, but rather represent the fitness costs of resistance to affect the transmission rate of pathogens from infectious to susceptible hosts (akin to colonization of empty patches in our model).

Models in McLean (1995) and Lipsitch (1997) also explore the potential for superinfection in models of evolved resistance to a prophylactic intervention. Nevertheless, the scope of our Models (2), (3), (4), (5), (6), (7), (8), (9) and their analyses differs subtly from that of McLean (1995) and Lipsitch (1997). For instance, the rate at which patches (hosts) become available for colonization in our models is proportional to the number of occupied patches, whereas McLean (1995)'s model assumes a constant flow of new hosts, and in Lipsitch (1997) it is the combined effect of cleared infections and the birth of new hosts that allows susceptible hosts (empty patches) to become available. As in the model of Lipsitch (1997) but unlike McLean (1995), Model (1) permits mutual superinfection. Consequently, the main focus of McLean (1995)'s model of superinfection is on a numerical analysis exploring how vaccination of a prevalent strain could select for a strain with a higher rate of spread. By contrast, we describe a survey across parameter space of how varying the potential for superinfection (competitive ability in our model) alters the ability of reduced prophylactic use to prevent the spread of the resistant strain in the long term. Furthermore, like McLean (1995) but unlike Lipsitch (1997), our model does not permit pathogen coexistence at a single site (or within a host). Our analyses also differ from Lipsitch (1997) in that we compare the dynamics of the spread of resistance under mutual superinfection and under unidirectional superinfection under the same framework in our comparisons of Models (1), (2). This enables us to highlight the effect of a competitive asymmetry in superinfections on the long‐term prevalence of resistant and antimicrobial‐sensitive strains (Figure 4) over different regions of parameter space than Lipsitch (1997). For instance, whereas Lipsitch (1997) find that use of a serotype or strain‐specific prophylactic consistently increases the prevalence of a nontarget type, we find that competitive asymmetry, particularly when antimicrobial use is constrained (analogous to low prophylactic coverage in Lipsitch (1997)) can prevent a resistant (nontarget) strain from spreading.

We note that despite some important parallels, the scope of our study as a whole differs from this earlier work by extending the analysis to include differentially connected pathogen habitats (or hosts). Below, we highlight some of the key implications of some of the main results from the analyses relaxing the assumption that every patch is equally accessible from every other patch. When the antimicrobial‐sensitive strain can persist in sites with the antimicrobial, we argued that the emergence of resistance is unlikely when there is a strong competitive asymmetry against the resistant strain and widespread antimicrobial use. This suggests that in the presence of strong fitness costs in environments without antimicrobials, the evolution of antimicrobial resistance itself may be somewhat limited even in the face of the large‐scale application of antimicrobials. Our results have implications for the debate over whether a trade‐off between antimicrobial resistance and competitive ability affects the emergence of resistance (Hughes & Andersson, 2015; Melnyk et al., 2015; Zur Wiesch, Engelstädter, & Bonhoeffer, 2010). The fact that, despite our results, antimicrobial resistance has been observed to evolve and spread across wide geographic regions may suggest that such fitness costs may not govern the evolution of resistance, at least on the temporal and spatial scales we modeled. In light of our analysis, we highlight applying our modeling strategy to systems where there is spatial heterogeneity in antimicrobial use as a particularly fruitful avenue in which to explore the role of a competition–resistance trade‐off in driving the dynamics of antimicrobial resistance.

The decisive role played by pleiotropy between resistance and migration is most apparent in metapopulations where patches vary in their connectivities. In such metapopulations, the effect of the competition–resistance trade‐off and restricting antimicrobial use on the spread of antimicrobial resistance strongly depends on the migration rate of the resistant strain relative to the sensitive strain. Increasing the migration rate has less of an effect in metapopulations where most patches are either highly connected or connected to one or two other patches. For pathogens moving through patches that vary considerably in their connectivities, understanding the nature of pleiotropy between antimicrobial resistance and colonization may be just as critical as understanding pleiotropy between competition and resistance in formulating effective responses. This result highlights the key role spatial structure can play in the spread of antimicrobial resistance, and the need to characterize the metapopulations in which resistant strains arise.

The scope of our analyses also differs most from work that focuses on well‐mixed host–pathogen systems when we extend our analyses to cases where a pathogen must alternate between treated and untreated habitats/hosts (Model (4), analogous to a scenario Park et al. (2015) note, but do not discuss or analyze further, where transmission is primarily between treated and untreated hosts). We showed that when the metapopulation consists of two distinct habitat types that the pathogen migrates between, the boundary equilibrium (8) of the antimicrobial‐sensitive strain is not viable when the fraction *f*_*n*_ of sites without the antimicrobial is large (and thus, pathogen dispersal is subject to a substantial bottleneck). This result suggests that when pathogens migrate between two types of sites, targeting antimicrobial use entirely to one type of patch may seem promising. However, our results show that this strategy is likely to be effective only if the pathogen undergoes limited dispersal, especially when the per‐patch competitive effects of antimicrobial‐sensitive strains on resistant strains are not very strong (e.g., Figures 3 and 5). Thus, targeting antimicrobial use to one kind of site is especially likely to be effective if there is a substantial trade‐off between competitive ability in the absence of the antimicrobial and resistance.

Model (4) and Model (3) can both describe situations where the direction of pathogen migration is more heterogenous among sites. However, contrasting our analyses of Model (3) with that of Model (4) shows how bottlenecking pathogen migration through sites with or without the antimicrobial can result in qualitatively distinct behavior in the spread of antimicrobial resistance through the metapopulation. When the pathogen migrates across several distinct types of patches (e.g., from highly connected clinical centers to less connected residences), this result underscores the value of targeting qualitatively distinct pathogen habitats for a given antimicrobial resistance management regime, rather than simply applying the same strategy uniformly across all types of patches.

In the interests of distilling the essential metapopulation‐level dynamics and maintaining tractability, we did not explicitly model the independent evolution of multiple resistant lineages across sites or the use of several complementary antimicrobials. Rather, we considered a situation where an initially spatially rare resistant mutant can expand across the landscape, and such a mutant cannot subsequently regain antimicrobial susceptibility. Our model could be modified to include multiple independent origins and possibly losses of antimicrobial resistance by explicitly modeling the patches occupied by distinct resistant lineages. In addition to the issues raised above, our model provides further points of departure that more detailed simulation models can address. By assuming all sites of a given type receive the antimicrobial (and thus the level of antimicrobial use to be constant) for the duration of the simulations, Model (4) cannot account for varying antimicrobial use temporally (e.g., Bergstrom et al., 2004). Modifying our models to account for temporal variability in antimicrobial use within the same site (e.g., Austin et al., 1997; Moghadas et al., 2008; Tanaka, Althouse, & Bergstrom, 2014), as well the use of alternative or complementary antimicrobials across sites (e.g., Bergstrom et al., 2004; Bonhoeffer et al., 1997; Saddler, Wu, Valckenborgh, & Tanaka, 2013), could provide insightful contrasts to the results we have presented here. In particular, we highlight two particularly fruitful directions of comparisons to our results. First, if evolved resistance to certain drugs increases sensitivity to other drugs (e.g., Baym et al., 2016; Imamovic & Sommer, 2013; Pál, Papp, & Lázár, 2015), this could be leveraged to alter the nature of pleiotropic fitness costs to resistance across habitats. Second, we feel a particularly promising direction of future work would be to compare and contrast the spatial mixing and cycling of antimicrobial use (as may occur, e.g., with Model 4) with a resistance management strategy based on temporal mixing and cycling (e.g., Bergstrom et al., 2004).

All of our models characterize situations where local epidemiological dynamics do not result in within‐patch cocirculation and coexistence of antimicrobial‐resistant and antimicrobial‐sensitive strains, at least over the timescale of metapopulation‐wide dynamics. In practice, antimicrobial‐sensitive and antimicrobial‐resistant strains may cocirculate locally, even over extended time horizons (e.g., Lipsitch, Colijn, Cohen, Hanage, & Fraser, 2009). For such coexistence to be feasible, antimicrobial‐sensitive and antimicrobial‐resistant strains must compete more strongly with themselves for hosts than with each other (Gause, 1934). This can occur if spatial structure is present (e.g., Amarasekare, 2003). Although our models provide a potential framework for understanding how antimicrobial‐resistant and antimicrobial‐susceptible strains can coexist on larger spatial scales, the fact that spatial structure can promote strain coexistence also highlights scenarios where our models are especially likely to apply. In particular, when the pathogen is likely to have a high local transmission rate, this can constrain the emergence of local spatial structure and reduce the prospects for long‐term strain coexistence. By contrast, if the pathogen spreads very slowly, when the patches themselves are highly heterogeneous internally and consist of several epidemiologically distinct host populations (e.g., large countries), then antimicrobial‐resistant and antimicrobial‐susceptible strains may become coendemic. Under such conditions, our models will need to be extended to include a category of patches where both strains may cocirculate over longer time horizons.

Similarly, by modeling patches as discrete entities, we do not explicitly consider the effects of within‐patch spatial heterogeneity in our analyses. Such within‐patch heterogeneity will give rise to a spatial gradient in antimicrobial concentrations, and the extent to which strains can replace each other across space becomes a dynamic process that depends on (potentially transient) local pathogen concentrations and the steepness of the spatial antimicrobial gradient. An important consequence of this is that the processes we predict to be key drivers of the emergence and spread of antimicrobial use across a pathogen metapopulation may not apply at finer spatial scales when pathogen habitats can no longer be treated as discrete, self‐contained units or when local dynamics happen on a comparable timescale as metapopulation dynamics. To explore the effect of these processes, explicitly tracking local epidemiological dynamics across a continuous spatial gradient (as in Smith et al., 2004 and Débarre et al., 2009), as well as approaches based on pair approximations (e.g.,. Sato, Matsuda, & Sasaki, 1994; Levin & Durrett, 1996; Bolker & Pacala, 1997) or even spatially explicit, agent‐based stochastic simulations and cellular automata (e.g., Hotchkiss, Strike, Simonson, Broccard, & Crooke, 2005; Murphy, Walshe, & Devocelle, 2008), may complement the patch‐occupancy‐based models we have proposed here to studying the spatial spread of antimicrobial resistance. Such detailed, stochastic simulations are needed to assess how implementing some of the strategies we explore (e.g., restraining migration or altering the global fitness costs) are likely to slow the spread of antimicrobial resistance in space under more realistic conditions.

Despite these simplifications, one advantage of our approach is that it can greatly facilitate linking theory and data. For instance, often the presence/absence of a pathogen at a particular site is easier to monitor than the local prevalence of infections, much less its epidemiological dynamics. When only presence/absence data are available, our model provides a potential point of departure for assessing whether antimicrobial resistance could spread across larger spatial scales.

Finally, we showed that reducing migration of the pathogen across the patches can have a large effect on constraining the emergence of resistant strains in the metapopulation. This is key, because antimicrobial treatments still play a vital role in reducing disease prevalence (e.g., Aminov, 2010; Gould & Bal, 2013). Our results also argue that the use of antimicrobials can be altered modestly, rather than drastically, to facilitate the competitive dominance of antimicrobial‐sensitive strains, at least when compensatory mutations (e.g., Handel et al., 2006) or other mitigating effects are absent. To be sure, even if the resistant strain's dispersal is constrained and it experiences a large competitive asymmetry, viz., the antimicrobial‐sensitive strain, under some conditions there is a clear advantage to reducing the spatial scale over which the antimicrobial is applied (e.g., Figure 1a). However, these advantages must be weighed against the very real costs of not treating host populations infected with antimicrobial‐sensitive pathogens with antimicrobials. If dispersal restrictions can delay the emergence of resistance, this means that more patches in our model would be able to use the antimicrobial when dispersal could be reduced. Such a strategy could complement strategies based on reducing antimicrobial use in space where the selective pressure is strong.

The result that constraining pathogen migration, either in addition to or to offset reduced antimicrobial use, has the potential to inform broader questions of how spatially based resistance management can be made more effective. Severely curtailing antimicrobial use in space (as explored in, e.g., Débarre et al., 2009) can be desirable from the perspective of reducing antimicrobial exposure and, thus, opportunities for resistance to emerge. Yet balancing the goals of preventing and reducing resistance against the benefits of widespread antimicrobial use remains challenging (e.g., Laxminarayan & Brown, 2001; Paul et al., 2006). Thus, given the prominent role antimicrobials continue to play in reducing the global infectious disease burden (e.g., Peter Davey, Wilcox, Irving, & Thwaites, 2015), there is a strong need for identifying strategies that may be just as effective as restricting antimicrobial use in preventing disease emergence. By examining the spread of antimicrobial resistance in a spatial context, our results help underscore that restricting antimicrobial use need not be the only approach to preventing antimicrobial resistance emergence across a landscape, particularly when the topology among sites varies. Nevertheless, restricting the distribution of toxic crops via refuge planting (Gould, 1994) has had a substantial effect on impeding the evolution of herbivorous resistance (Carriére, Crowder, & Tabashnik, 2010). Although developed in the context of antimicrobial resistance, our results suggest a meaningful role for dispersal barriers or shifting the pleiotropic costs, much as solutions developed to manage herbivore resistance via planting spatial refuges have also embraced such strategies. Using models that distill how the interplay between pathogen dispersal, local competition, and the spatial structure of the landscape drives the evolution of antimicrobial resistance, we hope our approach can meaningfully synthesize our understanding of antimicrobial resistance evolution and herbivore resistance management in spatially heterogenous systems.
