# Supplementary material for: Managing the emergence of pathogen resistance via spatially targeted antimicrobial use
Source: Evol Appl. 2018 Sep 26;11(10):1822–41. doi: 10.1111/eva.12683 (PMC6231480; doi:10.1111/eva.12683)
Supplement: Supplementary file 1 [file EVA-11-1822-s001.pdf]

# Supplementary Information S1 - Suppressing AMR when the susceptible strain is entirely excluded from sites where the antimicrobial is used

We compare the conditions necessary for antimicrobial resistance not to evolve in model (1). We characterize the ability of a pathogen carrying an AMR gene to increase when rare by deriving the conditions where the Jacobians of models (1), with $dn_S/dt \approx 0$, evaluated when $n_r(t), a_r(t) = 0$, have positive dominant eigenvalues. Suppression is therefore described as being possible when this quantity is negative.

In the absence of the susceptible strain being able to colonize sites with the antimicrobial, the dominant eigenvalue for the Jacobian of model (1) when the pathogen population is entirely antimicrobial susceptible is given by $\frac{1}{2}(\sqrt{\left((c_r + g_{r,s} - g_{s,r})\left(f_n - \frac{e_{n,s}}{c_s}\right) - c_r + e_{a,r} + e_{n,r}\right)^2 - \frac{4(c_r(c_s(f_n-1)(e_{n,r}+f_n g_{r,s})-e_{n,s}(e_{a,r}+(f_n-1)g_{r,s}))+e_{a,r}(c_s(e_{n,r}+f_n g_{r,s}-f_n g_{s,r})+e_{n,s}(g_{s,r}-g_{r,s})))}{c_s}} + \frac{e_{n,s}(c_r+g_{r,s}-g_{s,r})}{c_s} - (c_r(f_n-1)+e_{a,r}+e_{n,r}+f_n g_{r,s}-f_n g_{s,r}))$. Under biologically realistic conditions (i.e., all constants and state variables $> 0$), if the equilibrium frequency $n_s^\star$ of patches without the antimicrobial that are occupied by the susceptible strain is positive, the radicand in the eigenvalue is always positive and thus this eigenvalue is real. Under these same conditions, the dominant eigenvalue is positive whenever $e_{a,r} < (c_r(c_s + e_{n,s} - c_s f_n) + e_{n,s}(g_{r,s} - g_{s,r}))/c_s - (e_{n,r} + f_n(g_{r,s} - g_{s,r})$ as this renders the term outside the square root in the eigenvalue positive. If the term outside of the square root is negative (so that $e_{a,r} \geq (c_r(c_s + e_{n,s} - c_s f_n) + e_{n,s}(g_{r,s} - g_{s,r}))/c_s - (e_{n,r} + f_n(g_{r,s} - g_{s,r}))$, the dominant eigenvalue is negative if the square of the term outside of the square root exceeds the square of the radical. The difference between the squares is $4c_s(c_r(-e_{n,s}(e_{a,r} + (-1 + f_n)g_{r,s}) + c_s(-1 + f_n)(e_{n,r} + f_n g_{r,s}))+ e_{a,r}(e_{n,s}(-g_{r,s} + g_{s,r}) + c_s(e_{n,r} + f_n g_{r,s} - f_n g_{s,r})))$. When $e_{a,r} \geq (c_r(c_s + e_{n,s} - c_s f_n) + e_{n,s}(g_{r,s} - g_{s,r}))/c_s - (e_{n,r} + f_n(g_{r,s} - g_{s,r})$ (so that the real term of the eigenvalue is negative), this difference is positive if

$$
\begin{aligned}
g_{s,r} - g_{r,s} &< \frac{c_s e_{n,r}}{c_s f_n - e_{n,s}} \text{ and} \qquad \text{(S1-1)} \\
c_r &< \frac{e_{a,r}(c_s(e_{n,r} + f_n(g_{r,s} - g_{s,r})) + e_{n,s}(g_{s,r} - g_{r,s}))}{e_{n,s}(e_{a,r} + (f_n - 1)g_{r,s}) - c_s(f_n - 1)(e_{n,r} + f_n g_{r,s})},
\end{aligned}
$$

which is condition (5) of the main text. This therefore describes the conditions where the term outside of the square root is smaller than the radical when the term outside the square root is negative. Thus, the dominant eigenvalue as a whole is negative whenever these conditions hold.

If antimicrobial use is rare but the boundary equilibrium $n_s^\star$ is still viable (so that $f_n \to e_{n,s}/c_s$, and thus $n_s^\star = 1 - e_{n,s}/c_s$), $(c_s e_{n,r})/(c_s f_n - e_{n,s}) \to e_{n,r}/(1 - e_{n,s}/c_s) = e_{n,r}/n_s^\star$, i.e., the extinction rate of the resistant strain in sites without the antimicrobial, scaled by the equilibrium number of pathogen-occupied patches.
