# Supplementary material for: Managing the emergence of pathogen resistance via spatially targeted antimicrobial use
Source: Evol Appl. 2018 Sep 26;11(10):1822–41. doi: 10.1111/eva.12683 (PMC6231480; doi:10.1111/eva.12683)
Supplement: Supplementary file 2 [file EVA-11-1822-s002.pdf]

Supplementary Information S2 - Suppressing AMR when the susceptible strain can colonize sites where the antimicrobial is used

We characterize the conditions necessary for antimicrobial resistance to not evolve in model (2). Following the approach in Supplementary Information S1, we derive the conditions where the Jacobians of model (2), with $dn_S/dt, da_S/dt \approx 0$, evaluated when $n_r(t), a_r(t) = 0$, has a negative dominant eigenvalues (i.e., the resistant strain cannot invade).

If the susceptible strain can colonize sites with the antimicrobial, the dominant eigenvalue for the Jacobian of model (2) when the pathogen population is entirely antimicrobial susceptible is given by $\frac{1}{2}(\sqrt{(a_s^\star g_{r,s} + c_r(n_s^\star - 1) + e_{a,r} + e_{n,r} + g_{r,s} n_s^\star - g_{s,r} n_s^\star)^2 - 4(c_r((f_n - 1) g_{r,s}(a_s^\star + n_s^\star) + e_{a,r}(n_s^\star - f_n) + e_{n,r}(f_n - 1)) + e_{a,r}(a_s^\star g_{r,s} + e_{n,r} + n_s^\star(g_{r,s} - g_{s,r})))} - a_s^\star g_{r,s} + c_r(-n_s^\star) + c_r - e_{a,r} - e_{n,r} - g_{r,s} n_s^\star + g_{s,r} n_s^\star)$.

Under biologically realistic conditions (i.e., all constants and state variables > 0), if $n_S^\star < f_n$ and $a_S^\star < 1 - f_n$, this eigenvalue is real and, following the argument in Supplementary Information S1, the dominant eigenvalue is positive whenever $e_{a,r} < (c_r(c_s + e_{n,s} - c_s f_n) + e_{n,s}(g_{r,s} - g_{s,r}))/c_s - (e_{n,r} + f_n(g_{r,s} - g_{s,r}))$. If the term outside of the square root is negative (so that $e_{a,r} < (c_r(c_s + e_{n,s} - c_s f_n) + e_{n,s}(g_{r,s} - g_{s,r}))/c_s - (e_{n,r} + f_n(g_{r,s} - g_{s,r})))$, the dominant eigenvalue is positive if, and only if, the square of the radical exceeds the square of the real term. As in Supplementary Inforamtion S1 we take the the difference between the squares; here, this quantity is given by $-4(e_{a,r}(e_{n,r} + a_s^\star g_{r,s} + (g_{r,s} - g_{s,r}) n_s^\star) + c_r(e_{n,r}(-1 + f_n) + (-1 + f_n) g_{r,s}(a_s^\star + n_s^\star) + e_{a,r}(-f_n + n_s^\star)))$. This difference is negative (i.e., the dominant eigenvalue as a whole is negative) whenever condition (7) of the main text holds.

Condition (7) of the main text also implies $c_r < -\frac{e_{a,r}(a_s^\star g_{r,s} + e_{n,r} + n_s^\star(g_{r,s} - g_{s,r}))}{(f_n - 1) g_{r,s}(a_s^\star + n_s^\star) + e_{a,r}(n_s^\star - f_n) + e_{n,r}(f_n - 1)}$. When $f_n \to 0$ and $n_s^\star \to 0$, the expression on the right hand side approaches $e_{a,r}$, as described in the main text.
