# Supplementary material for: Managing the emergence of pathogen resistance via spatially targeted antimicrobial use
Source: Evol Appl. 2018 Sep 26;11(10):1822–41. doi: 10.1111/eva.12683 (PMC6231480; doi:10.1111/eva.12683)
Supplement: Supplementary file 3 [file EVA-11-1822-s003.pdf]

Supplementary Information S3 - Suppressing AMR when the pathogen must migrate between two patch types and when the susceptible strain can colonize sites where the antimicrobial is used

We characterize the conditions necessary for antimicrobial resistance to not evolve in model (4). We derive the conditions where the Jacobian of model (3), with $dn_S/dt, da_S/dt \approx 0$, evaluated when $n_r(t), a_r(t) = 0$, has a negative dominant eigenvalue.

If the susceptible strain can colonize sites with the antimicrobial, the dominant eigenvalue for the Jacobian of model (4) when the pathogen population is entirely antimicrobial susceptible is given by $\frac{1}{2}\left(\sqrt{(a_s^\star g_{r,s} - e_{a,r} + e_{n,r})^2 - 4c_r(f_n - 1)(c_r f_n - c_r n_s^\star + g_{s,r} n_s^\star)} - a_s^\star g_{r,s} - e_{a,r} - e_{n,r}\right)$. Under biologically realistic conditions (i.e., all constants and state variables > 0), the conditions for the dominant eigenvalue to be positive reduce to $g_{s,r} > \frac{(c_s f_n + e_{a,s})\left(c_s e_{n,s}\left(c_r^2(f_n-1)f_n + e_{a,r}e_{n,r}\right) + c_r^2 e_{a,s}e_{n,s}(f_n-1) - c_s^2 e_{a,r}e_{n,r}(f_n-1)\right) + e_{a,r}g_{r,s}(c_s(f_n-1)-e_{n,s})\left(c_s^2(f_n-1)f_n + e_{a,s}e_{n,s}\right)}{c_r(f_n-1)(c_s f_n + e_{a,s})(c_s^2(f_n-1)f_n + e_{a,s}e_{n,s})}$. Under these conditions, the radicand is positive and the dominant eigenvalue is also real. Furthermore, as $e_{a,r} \to 0$ and $f_n \to 0$, this condition reduces to the requirement that $g_{s,r} > c_r(c_s + e_{a,s})/e_{a,s}$, as described in the main text.

By contrast, if $\frac{\left(c_s^2 f_n - c_s(c_s + e_{n,s})\right)\left(\frac{4cr^2 e_{n,s}(f_n-1)(c_s f_n + e_{a,s})}{c_s^2 f_n - c_s(c_s+e_{n,s})} + \left(\frac{g_{r,s}\left(c_s^2(f_n-1)f_n + e_{a,s}e_{n,s}\right)}{c_s(c_s f_n + e_{a,s})} + e_{a,r} - e_{n,r}\right)^2\right)}{4cr(f_n-1)(c_s^2(f_n-1)f_n + e_{a,s}e_{n,s})} \leq g_{s,r} < -\frac{\left(c_s^2 f_n - c_s(c_s+e_{n,s})\right)\left(\frac{cr^2 e_{n,s}(f_n-1)(c_s f_n + e_{a,s})}{c_s(c_s(-f_n)+c_s+e_{n,s})} + e_{a,r}\left(e_{n,r} - \frac{g_{r,s}\left(c_s^2(f_n-1)f_n + e_{a,s}e_{n,s}\right)}{c_s(c_s f_n + e_{a,s})}\right)\right)}{cr(f_n-1)(c_s^2(f_n-1)f_n + e_{a,s}e_{n,s})}$, the dominant eigenvalue is negative (and also real). Substituting the antimicrobial-sensitive boundary equilibrium $(a_s^\star, n_s^\star)$ from eq. 8 in the main text into this last inequality yields condition (9) of the main text.
