# Supplementary material for: Managing the emergence of pathogen resistance via spatially targeted antimicrobial use
Source: Evol Appl. 2018 Sep 26;11(10):1822–41. doi: 10.1111/eva.12683 (PMC6231480; doi:10.1111/eva.12683)
Supplement: Supplementary file 4 [file EVA-11-1822-s004.pdf]

Supplementary Figure S4 - The outcome of an eco-evolutionary interaction between the evolution of antimicrobial resistance and the pathogen's metapopulation dynamics when the tradeoff between competitive ability and resistance is severe.

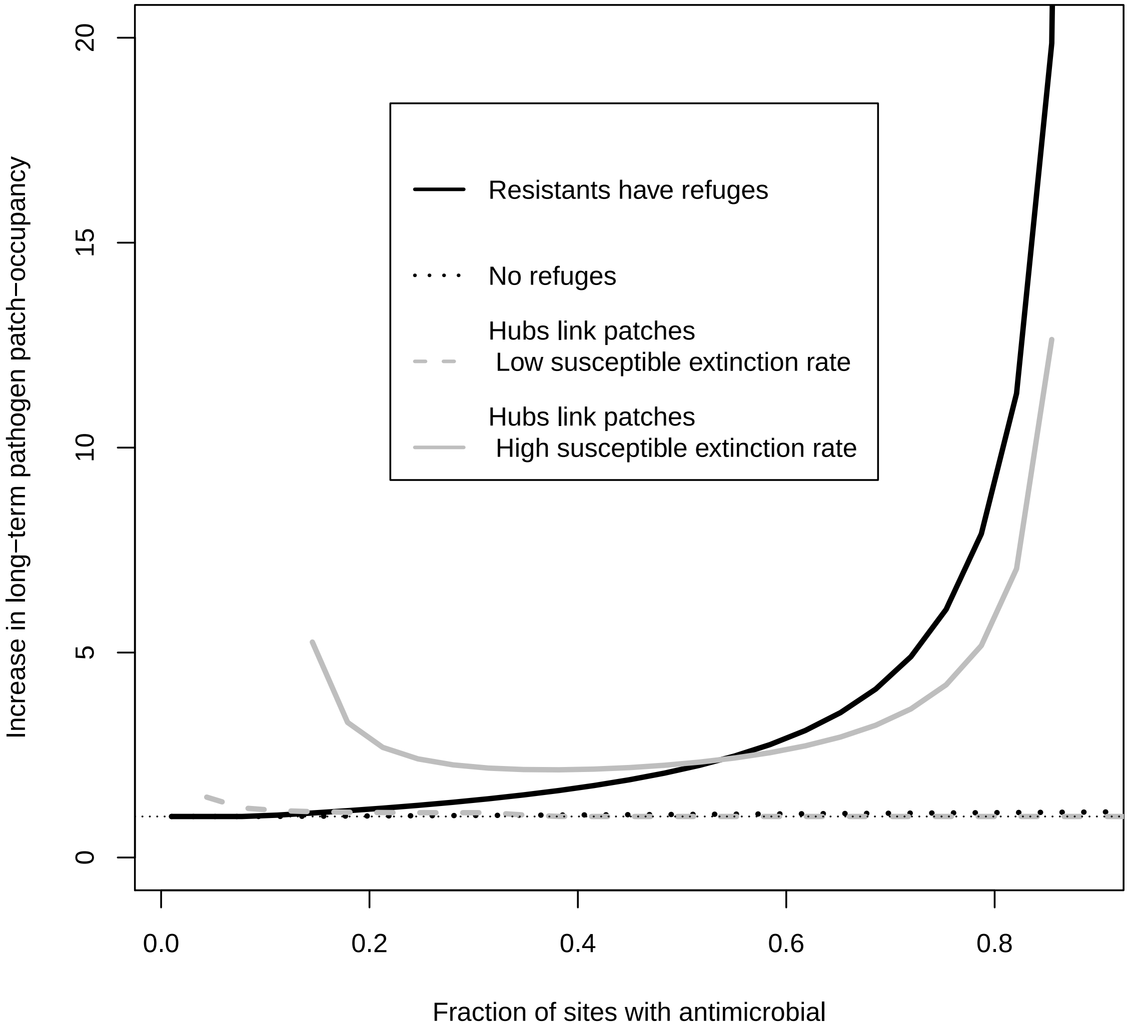

Figure 1: The outcome of an eco-evolutionary interaction between the evolution of antimicrobial resistance and the pathogen's metapopulation dynamics, in the presence of a relatively sever cost of resistance. The vertical axis represents the factor by which the equilibrium number of patches occupied across the landscape increases in response to the emergence of antimicrobial resistance. For model (2-3), the susceptible extinction rate $e_{a,s}$ in patches with the antimicrobial was varied from $e_{a,s} = 2e_{n,s}$ to $e_{a,s} = 10e_{n,s}$; unlike in the main text, the plotted values here are for the cases where $g_{r,s} = 10g_{s,r}$, implying the susceptible strain can replace the resistant strain at an order of magnitude higher per-site rate. All other parameter values are as in Figure 1.
